# Supplementary material for: Chameleon-inspired tunable multi-layered infrared-modulating system via stretchable liquid metal microdroplets in elastomer film
Source: Nat Commun. 2024 Jun 26;15:5395. doi: 10.1038/s41467-024-49849-y (PMC11208509; doi:10.1038/s41467-024-49849-y)
Supplement: Supplementary file 1 — Supplementary Information [file 41467_2024_49849_MOESM1_ESM.pdf]

Supplementary Information

Chameleon-Inspired Tunable Multi-layered Infrared-Modulating System via Stretchable Liquid Metal Microdroplets in Elastomer Film

Yingyue Zhang,[1,2] Hanrui Zhu,[1,2] Shun An,[1,2] Wenkui Xing,[1,2] Benwei Fu,[1,2] Peng Tao,[1,2] Wen Shang,[1,2] Jianbo Wu,[1,2] Michael D. Dickey,[3]* Chengyi Song,[1,2]* and Tao Deng,[1,2]*

[1]The State Key Laboratory of Metal Matrix Composites, School of Materials Science and Engineering, Shanghai Jiao Tong University, 800 Dong Chuan Road, Shanghai 200240, P.R. China

[2]Center of Hydrogen Science, Shanghai Jiao Tong University, 800 Dong Chuan Road, Shanghai 200240, P.R. China

[3]Department of Chemical and Biomolecular Engineering, North Carolina State University, 911 Partners Way, Raleigh, NC 27695, USA

*Corresponding author. **E-mail**: chengyi2013@sjtu.edu.cn; mddickey@ncsu.edu; dengtao@sjtu.edu.cn

This PDF file includes:

Calculation of mechanical actuation
Calculation of infrared spectroscopy
Simulation of the liquid metal droplets' deformation
Supplementary Figures 1 to 34
Supplementary Tables 1 to 11
Supplementary References

Other Supplementary Materials for this manuscript include the following:

Supplementary Movies 1 to 5

Calculation of mechanical actuation

The samples were stretched by hand and fixed in an acrylic ring for further tests. The areal strain was used in this work to measure the deformation of the samples. The areal strains were calculated according to Equation (S1):

$$areal\ strain(\%) = \left[\frac{A_1 - A_0}{A_0}\right] \times 100\% \qquad \text{(S1)}$$

where A_0 is the area of the sample before stretching, and A_1 is the area of the sample after stretching[1].

Calculation of infrared spectroscopy

The total reflectance and total transmittance of bilayered Ecoflex/EGaIn (BLEE) films (Ecoflex/EGaIn mass ratios: 1:0, 1:2, 1:4, 1:6, 1:8, and 1:10) under different areal strains (0%, 137%, 525%, 1011%, and 1500%) were characterized by FTIR, and Pike Technologies Diffuse Gold Standard was used as a reference. The total absorptance can be calculated by Equation (S2)[2]:

$$total\ absorptance(\%) = 100\% - total\ reflectance(\%) - total\ transmittance(\%) \qquad \text{(S2)}$$

The specular reflectance of samples under specific areal strains was directly characterized by an infrared imaging microscope because the detector of an infrared imaging microscope only received the light from samples, which satisfied specular reflectance. In the above measurements, specular reflectance and total reflectance have the following relationship:

$$total\ reflectance(\%) = specular\ reflectance(\%) + diffuse\ reflectance(\%) \qquad \text{(S3)}$$

According to Planck's formula:

$$M(\lambda, T) = \frac{2\pi hc^2}{\lambda^5} \times \frac{1}{e^{\frac{hc}{\lambda kt}} - 1} \qquad \text{(S4)}$$

where h is Planck constant (6.626×10^{-34} J·s), c is the speed of light (3×10^{8} m/s) and k is Boltzmann constant (1.38×10^{-23} J/K). And λ is the wavelength, T is the temperature. According to the above measurements, the average reflectance ($R_{average}$) within the range of 7.5 μm to 14 μm (the wavelength of the infrared (IR) camera) can be calculated with the following Equation (S5).

$$R_{average} = \frac{\int_{7.5\ \mu m}^{14\ \mu m} R(\lambda) \times M(\lambda) d\lambda}{\int_{7.5\ \mu m}^{14\ \mu m} M(\lambda) d\lambda} \qquad \text{(S5)}$$

where $R(\lambda)$ is the total reflectance measured with FTIR and $M(\lambda)$ is from Equation (S4) when T is equal to 301 K (room temperature). Besides that, the average absorptance ($A_{average}$) and average transmittance ($T_{average}$) can be calculated in the same method. IR camouflage properties can be also predicted from the measured results of reflectance.

$$A_{camera} \times E(T_{camera}) = T'_{sample} \times E(T_{hot\,plate}) + A_{sample} \times E(T_{sample}) + R_{sample} \times E(T_{environment}) \quad \text{(S6)}$$

$$\int_{7.5\,\mu m}^{14\,\mu m} M(\lambda, T) d\lambda = E(T) \quad \text{(S7)}$$

where A_{camera} is emissivity of IR camera, which is set to be 1. T_{camera} is the temperature shown in IR camera. T'_{sample}, A_{sample} and R_{sample} are the $T_{average}$, $A_{average}$ and $R_{average}$ of samples, respectively. $T_{hot\,plate}$, T_{sample} and $T_{environment}$ are the temperature of the hot plate, sample and environment (301 K), respectively. Because of the low thermal impedance (~3.08×10^{-5} Km^2/W) of the sample, we assumed that T_{sample} is equal to $T_{hot\,plate}$ (349 K). Then, the Equation (S6) can be further written in the following way:

$$A_{camera} \times E(T_{camera}) = (1 - R_{sample}) \times E(T_{hot\,plate}) + R_{sample} \times E(T_{environment}) \quad \text{(S8)}$$

T_{camera} can be obtained from the spectral results and the calculations. The calculations were performed with code developed in MATLAB.

Especially, for the sample made from 70 ℃- melting point (mp) alloy, the $T_{environment}$ is 295 K. And the $T_{hot\,plate}$ are set to be 337 K and 377 K, respectively.

Simulation of the liquid metal droplets' deformation

We used COMSOL Multiphysics to simulate the deformation of liquid metal droplets with different droplet sizes within Ecoflex matrix when applying 1500% areal strain. We defined the deformation of liquid metal droplets as *(R`-R)/R*. *R* was the radius of liquid metal droplets without areal strain and *R`* was the radius of liquid metal droplets with areal strain of 1500% as illustrated in Supplementary Fig. 11a. Young's modulus of Ecoflex matrix (E_0) was set to 0.102 MPa as

measured in Supplementary Table 3. Poisson's ratio of Ecoflex matrix (V_0) was set to 0.4287[3]. Effective Young's modulus (E_{eff}) of liquid metal droplet was calculated by Equation (S9)[4]:

$$E_{eff} = \frac{E_i t}{2R}\left(\frac{5E_i t + 2E_0 R(7-5V_i)}{E_i t(7+5V_i) + 10E_0 R\left(1-V_i^2\right)}\right) \tag{S9}$$

where the Young's modulus of gallium oxide E_i was set to be 30 GPa[5]. Poisson's ratio of gallium oxide (V_i) was set to 0.3[6]. The thickness of the gallium oxide layer (t) was 1.4 nm according to the transmission electron microscope (TEM) results in Supplementary Fig. 13. We simulated the deformation of liquid metal droplets with diameters from 0.1 μm to 40 μm under applied areal strain of 1500%, and the theoretical calculation results were shown in Supplementary Figs. 11 (b and c).

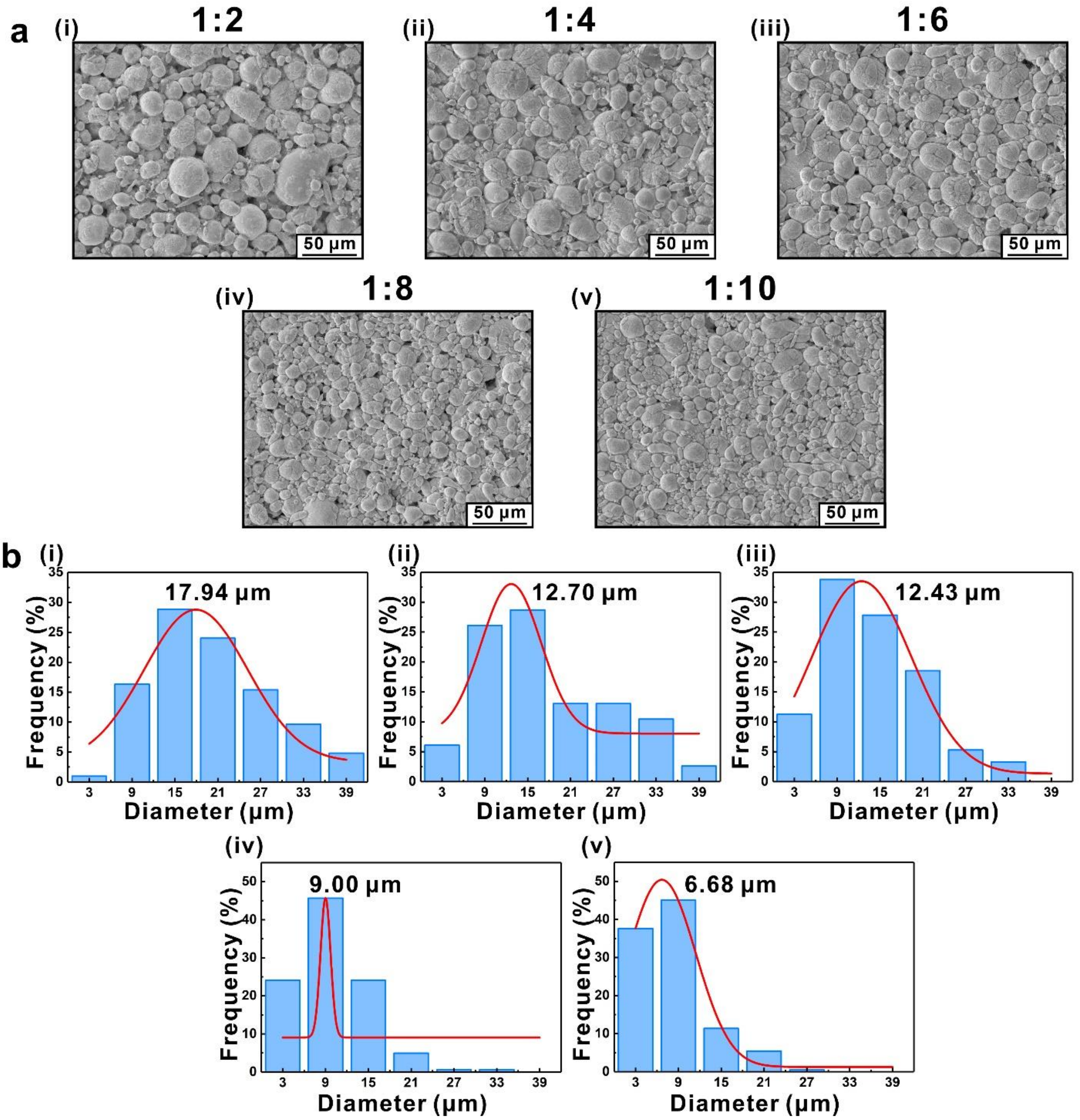

Supplementary Figure 1. **Characterizations of liquid metal droplets. a** The scanning electron microscopy (SEM) images (the scale bar is 50 μm) and **b** size distributions of liquid metal droplets extracted from liquid metal-based elastomer composites with different mass ratios of Ecoflex/EGaIn (1:2, 1:4, 1:6, 1:8, and 1:10).

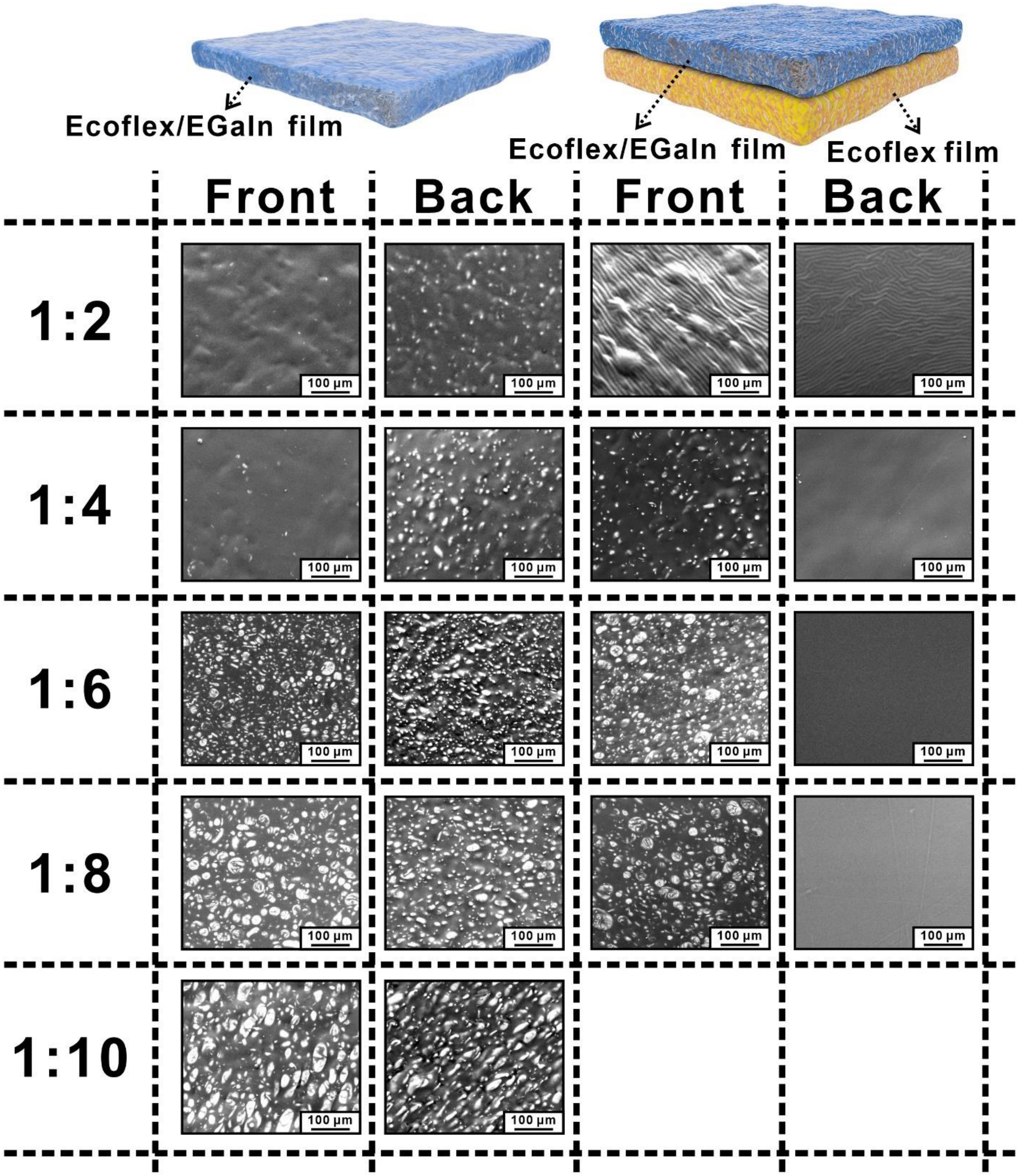

Supplementary Figure 2. SEM images of single-layered and BLEE films. The SEM images of single-layered and BLEE films with different mass ratios of Ecoflex/EGaIn (1:2, 1:4, 1:6, 1:8, and 1:10). The scale bar is 100 µm.

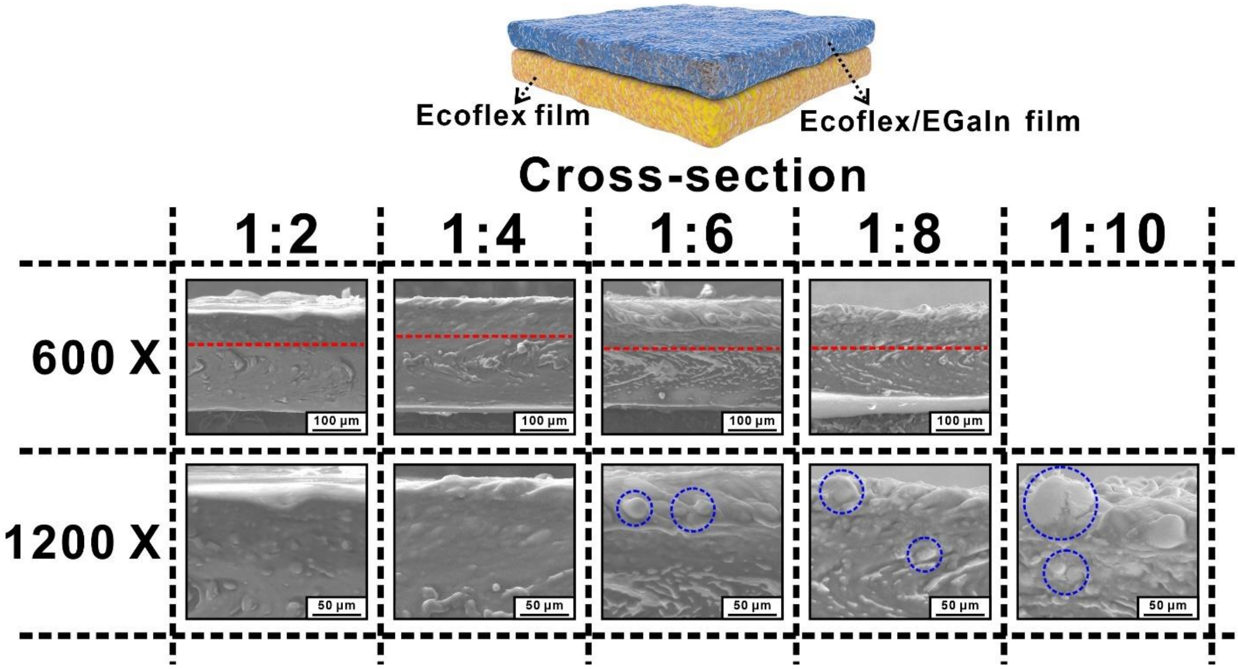

Supplementary Figure 3. SEM images of the cross-section of BLEE films. The SEM images under different magnifications (600× and 1200×) of BLEE films prepared by different mass ratios of Ecoflex/EGaIn (1:2, 1:4, 1:6, 1:8, and 1:10). The pure Ecoflex layer was under the red dashed line and the Ecoflex/EGaIn layer was above the red dashed line. Liquid metal droplets were marked with blue dashed circles. The scale bar for the images with a magnification of 600× is 100 μm. And the scale bar for the images with a magnification of 1200× is 50 μm.

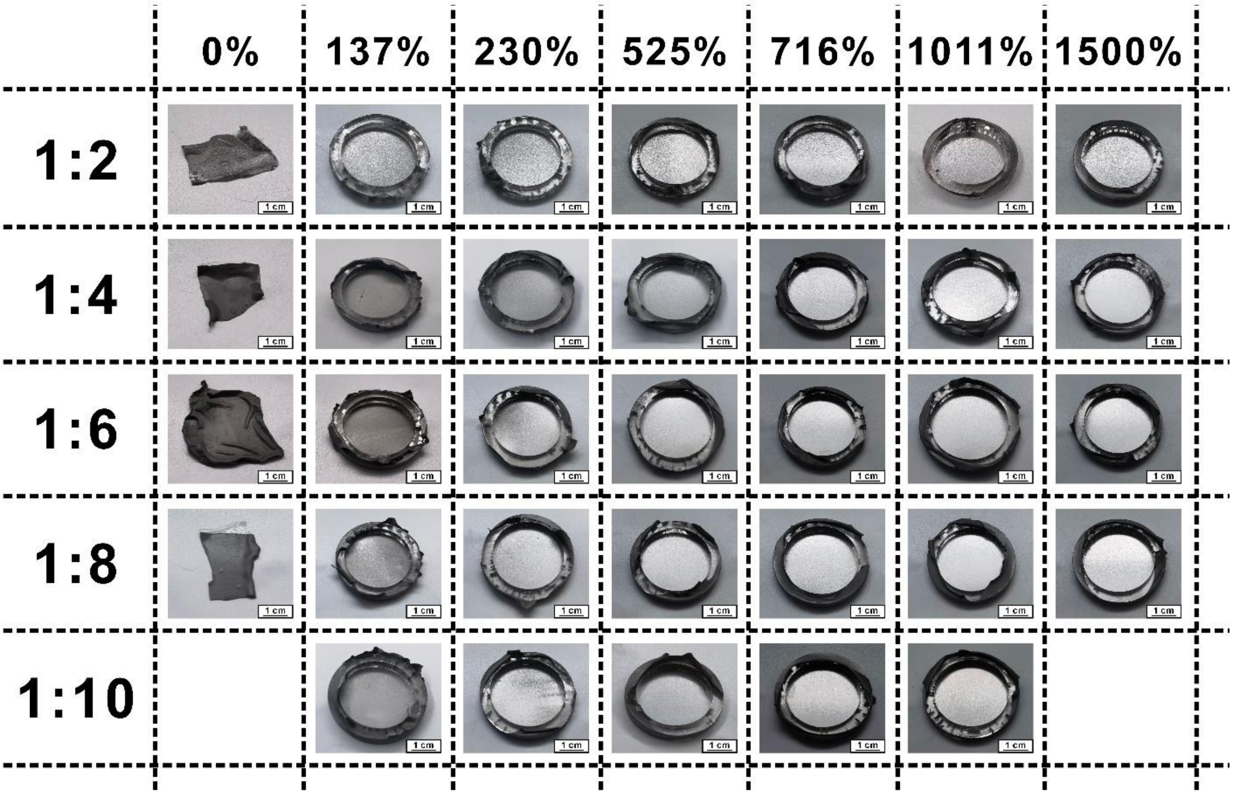

Supplementary Figure 4. Optical images of BLEE films. Optical images of BLEE films with different mass ratios of Ecoflex/EGaIn (1:2, 1:4, 1:6, 1:8, and 1:10), and stretched under different areal strains (0%, 137%, 230%, 525%, 716%, 1011%, and 1500%). The optical images were taken by an optical camera. The scale bar is 1 cm.

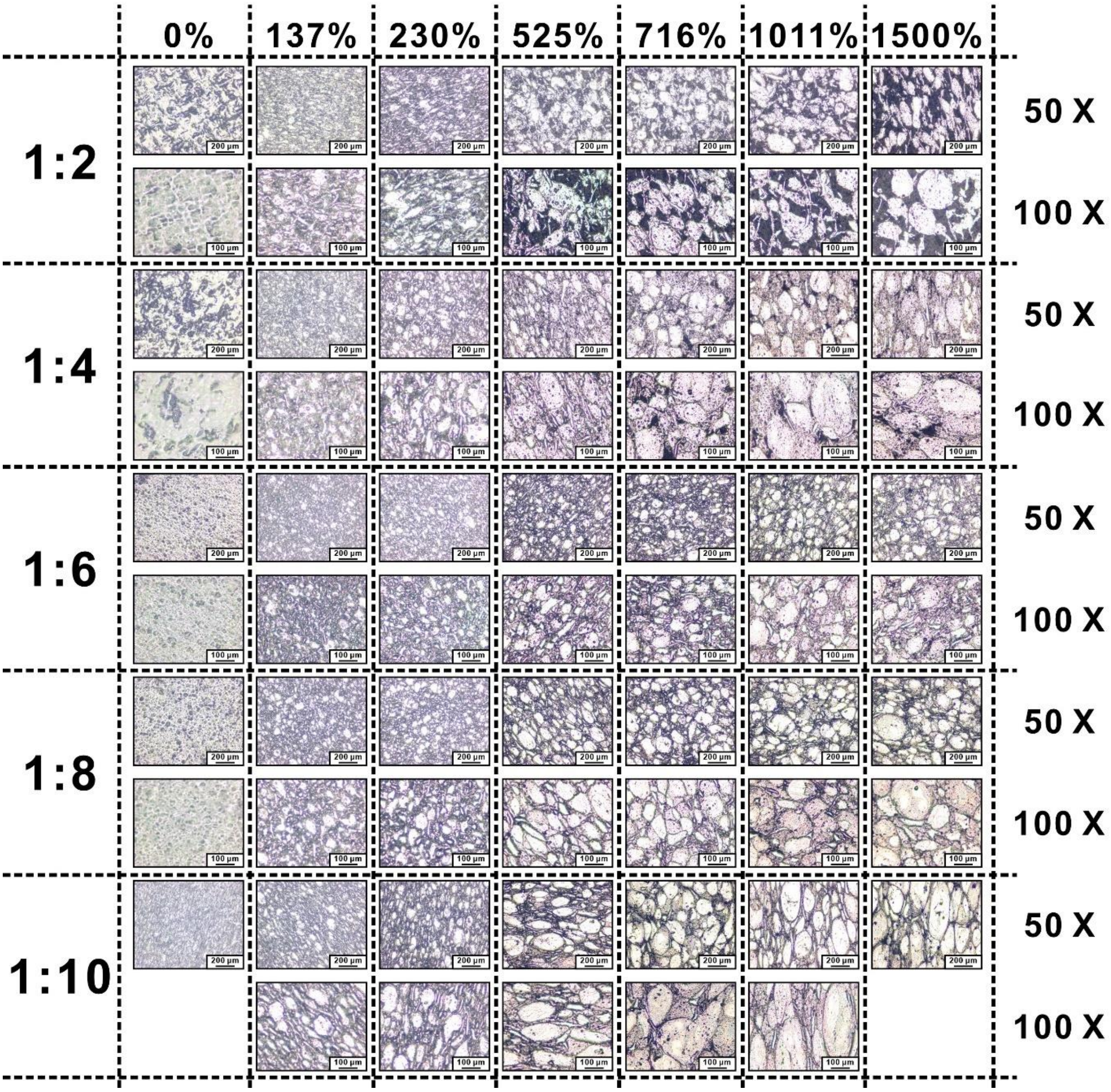

Supplementary Figure 5. Optical images of BLEE films. Optical images of BLEE films with different mass ratios of Ecoflex/EGaIn (1:2, 1:4, 1:6, 1:8, and 1:10), and are stretched under different areal strains (0%, 137%, 230%, 525%, 716%, 1011%, and 1500%). The optical images were taken by optical microscope. For each mass ratio of BLEE films, two kinds of magnifications (50× and 100×) were used. The scale bar for the images with a magnification of 50× is 200 μm. And the scale bar for the images with a magnification of 100× is 100 μm.

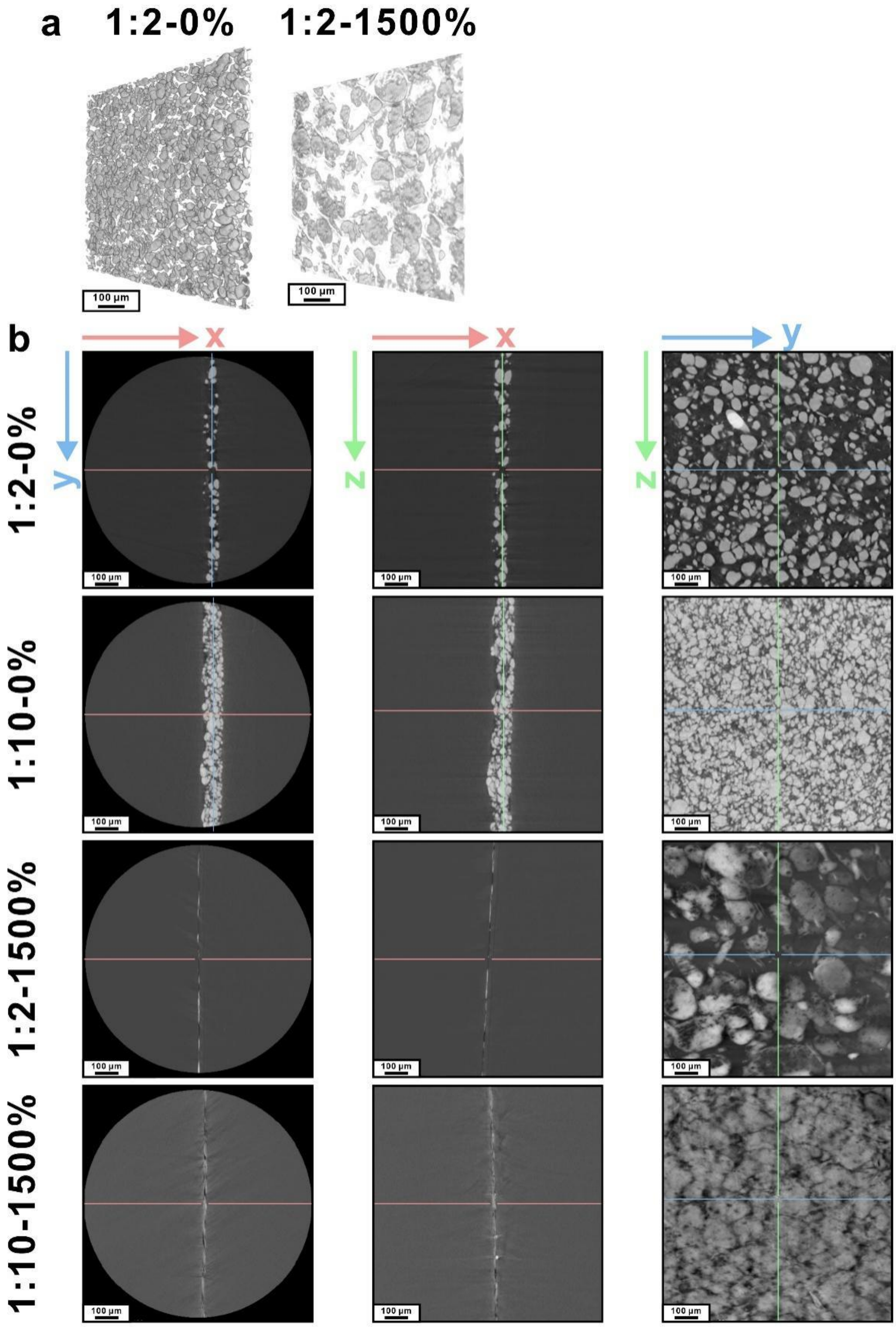

Supplementary Figure 6. X-ray microscope (XRM) results of BLEE films. a The three-dimensional (3D) XRM results and **b** the two-dimensional cross-section results of BLEE films with mass ratios 1:2 and 1:10 before and after applying areal strain (1500%). For the stretched BLEE films, the position of the y axle in the xy cross-section and the z axle in the xz cross-section coincide with the sample plane. The scale bar is 100 μm.

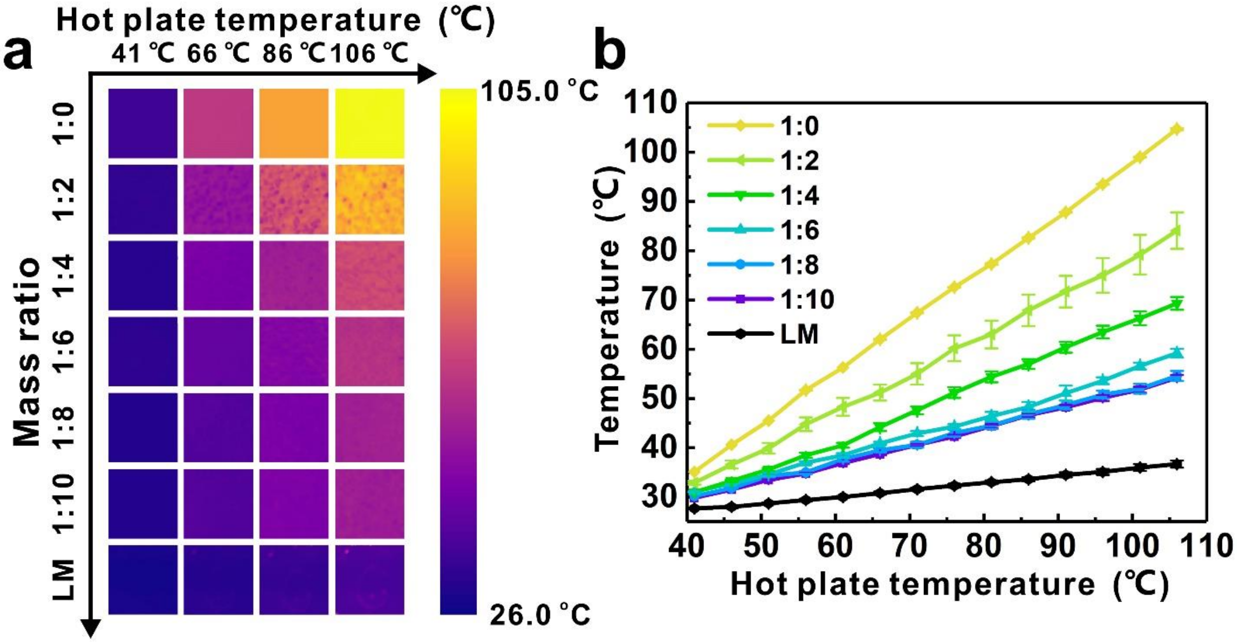

Supplementary Figure 7. IR camouflage properties of BLEE films and pure EGaIn. BLEE films with different mass ratios of Ecoflex/EGaIn (1:2, 1:4, 1:6, 1:8, and 1:10) after applying areal strain of 1500%, and pure EGaIn. These samples were all placed on the hot plate with different temperatures (41 °C, 66 °C, 86 °C, and 106 °C). **a** The IR images of BLEE films. **b** The temperature curve of each sample as a function of hot plate temperature and temperature data are taken from IR images of Supplementary Fig. 7a. All error bars represent the standard deviation.

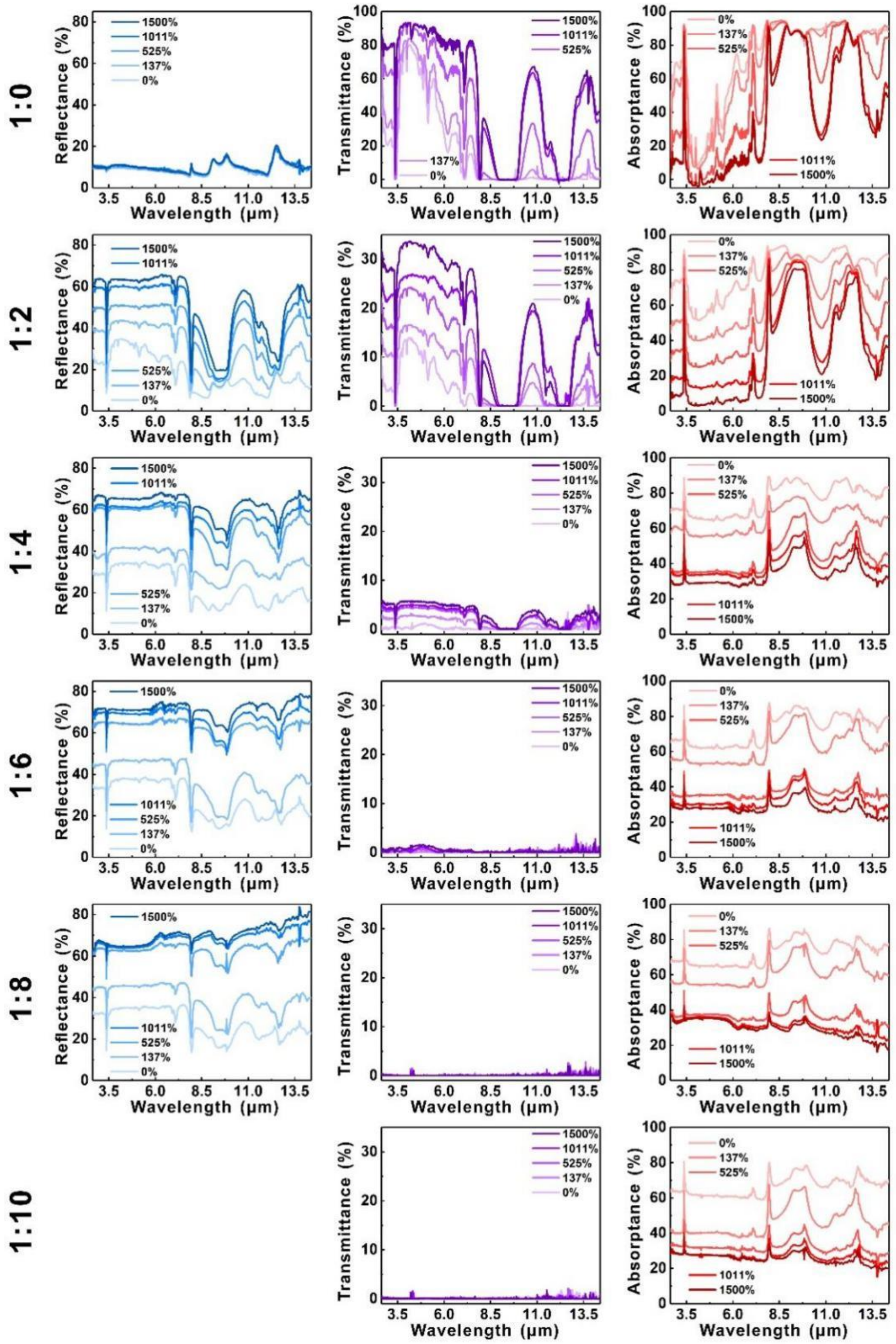

Supplementary Figure 8. The reflectance, transmittance, and absorptance spectra of BLEE films. The reflectance, transmittance, and absorptance spectra of BLEE films with different mass ratios of Ecoflex/EGaIn (1:2, 1:4, 1:6, 1:8, and 1:10) after applying different areal strains (0%, 137%, 525%, 1011%, and 1500%).

Supplementary Note: Films with low mass ratios (e.g.1:2 and 1:4) increase transmittance when the films are thin by stretching. However, when the mass ratio increases to 1:8 or 1:10, the transmittance approaches zero with or without stretching. It is attributed that the amount of EGaIn droplets in such films reach a threshold and no gaps are left between neighbored droplets.

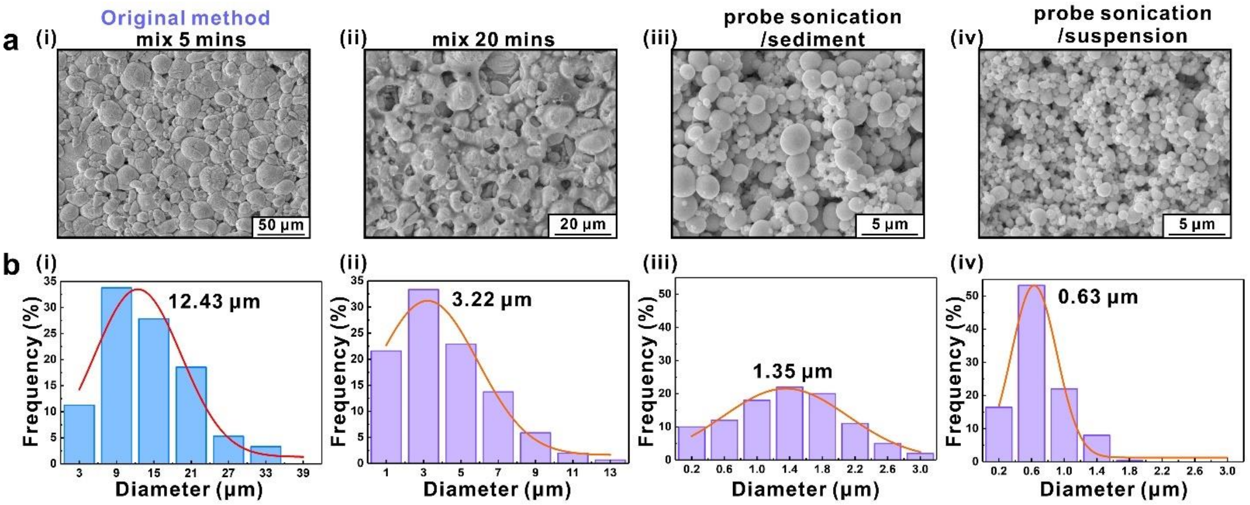

Supplementary Figure 9. Characterizations of liquid metal droplets. a The SEM images (the scale bars are (i) 50 μm, (ii) 20 μm, (iii) 5 μm, and (iv) 5 μm) and **b** size distributions of liquid metal droplets in BLEE films with a mass ratio of 1:6 prepared by different methods ((i) mix 5 mins, (ii) mix 20 mins, (iii) probe sonication/sediment, and (iv) probe sonication/suspension). (Supplementary Figs. 9a (i) and b (i) were taken from Supplementary Figs. 1a (iii) and b (iii).)

Supplementary Note 1: To clarify the influence of the size of liquid metal droplets within Ecoflex on IR reflectance, we fixed the Ecoflex/EGaIn mass ratio of 1:6 and prepared 4 samples by different methods.

Sample #1: mix 5 mins: we mixed liquid metal and Ecoflex for 5 mins and prepared the BLEE with an average liquid metal droplet size of 12.43 μm (Supplementary Fig. 9 (i)), which followed the same method in the manuscript.

Sample #2: mix 20 mins: we extended the mechanical mixing time of liquid metal and Ecoflex from 5 mins in the manuscript to 20 mins, and prepared the BLEE with an average liquid metal droplet size of 3.22 μm (Supplementary Fig. 9 (ii)), which is smaller than the sizes in manuscript (as shown in Supplementary Fig. 9 (i)).

Sample #3: probe sonication/sediment: we further used probe sonication to prepare liquid metal nanoparticles. The liquid metal was sonicated for 15 mins in ethanol with a power of 300 W. The above solution was centrifuged at 16 g for 10 minutes. The sediment was collected and used to prepared BLEE film with an average liquid metal droplet size of 1.35 μm (Supplementary Fig. 9 (iii)).

Sample #4: probe sonication/suspension: the above suspension was further centrifugated. The particles with an average liquid metal droplet size of 0.63 μm were collected and used to prepare BLEE film (Supplementary Fig. 9 (iv)).

Supplementary Note 2: The above as-prepared liquid metal droplets from either probe sonication or centrifugation were further transferred into hexane solution, and purified twice with 409 g under centrifugation for 10 mins before being used to prepare BLEE films.

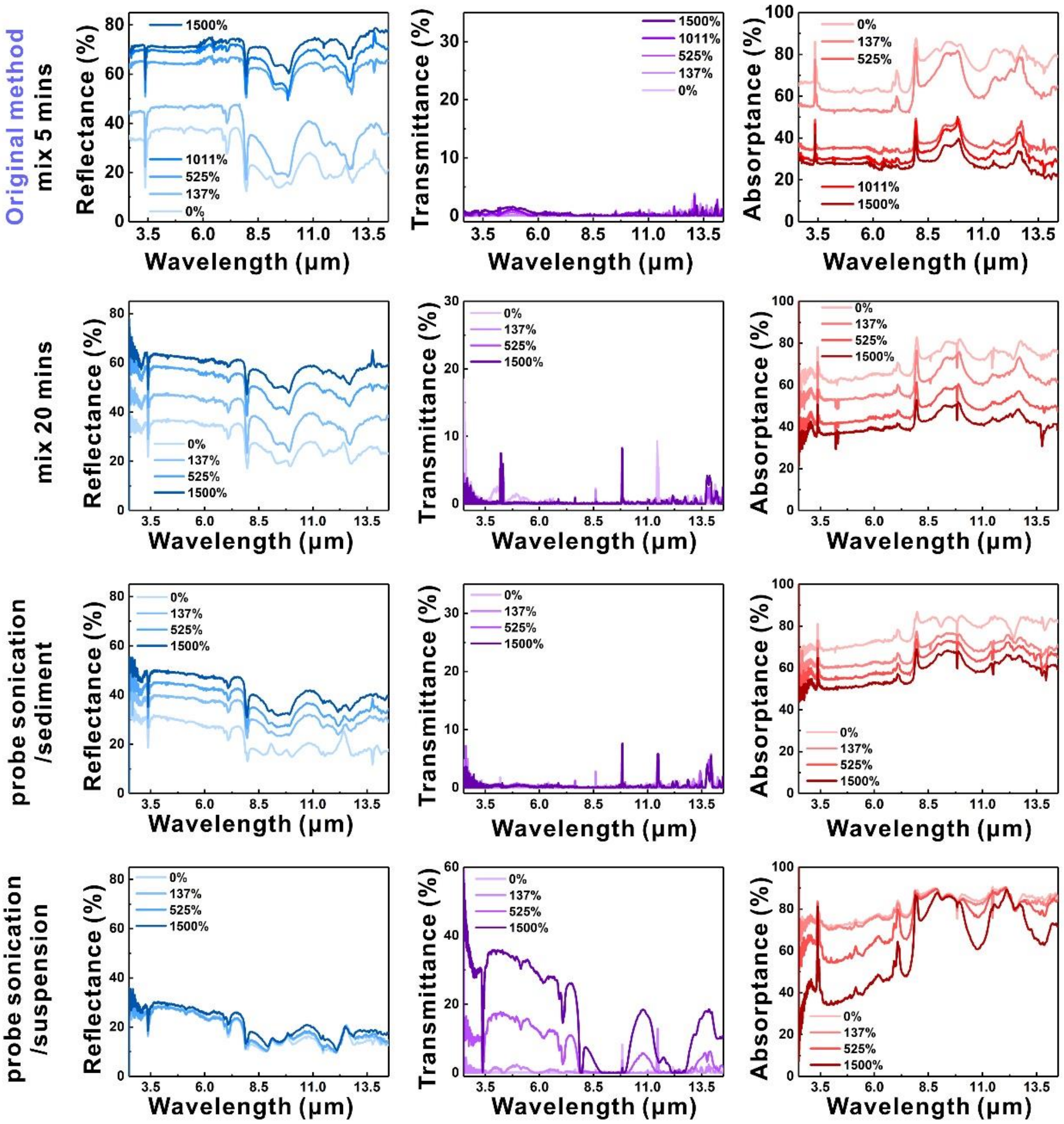

Supplementary Figure 10. The reflectance, transmittance, and absorptance spectra of BLEE films. The reflectance, transmittance, and absorptance spectra of BLEE films prepared by different liquid metal droplet sizes after applying different areal strains (0%, 137%, 525%, and 1500%). (Figures of sample "mix 5 mins" were taken from Supplementary Fig. 8.)

Supplementary Note: From the SEM and IR specular reflectance analysis (Supplementary Figs. 9 and 10), we observed that BLEE films prepared by "Sample #1: mix 5 mins", "Sample #2: mix 20 mins" and "Sample #3: probe sonication/sediment" showed IR reflectance changes with different areal strains. However, the IR reflectance spectra of BLEE film prepared by "Sample #4: probe sonication/suspension" did not show obvious change under areal strain. It is attributed that the applied external force should be larger than $2\gamma/R$ for triggering the liquid metal particle deform according to the Young-Laplace equation, in which γ is the surface tension of liquid metal (0.624 N/m) and R is the radius of the liquid metal particle. As for the liquid metal nanodroplets, the applied external force becomes very high so the deformation of liquid metal nanodroplets becomes difficult[7].

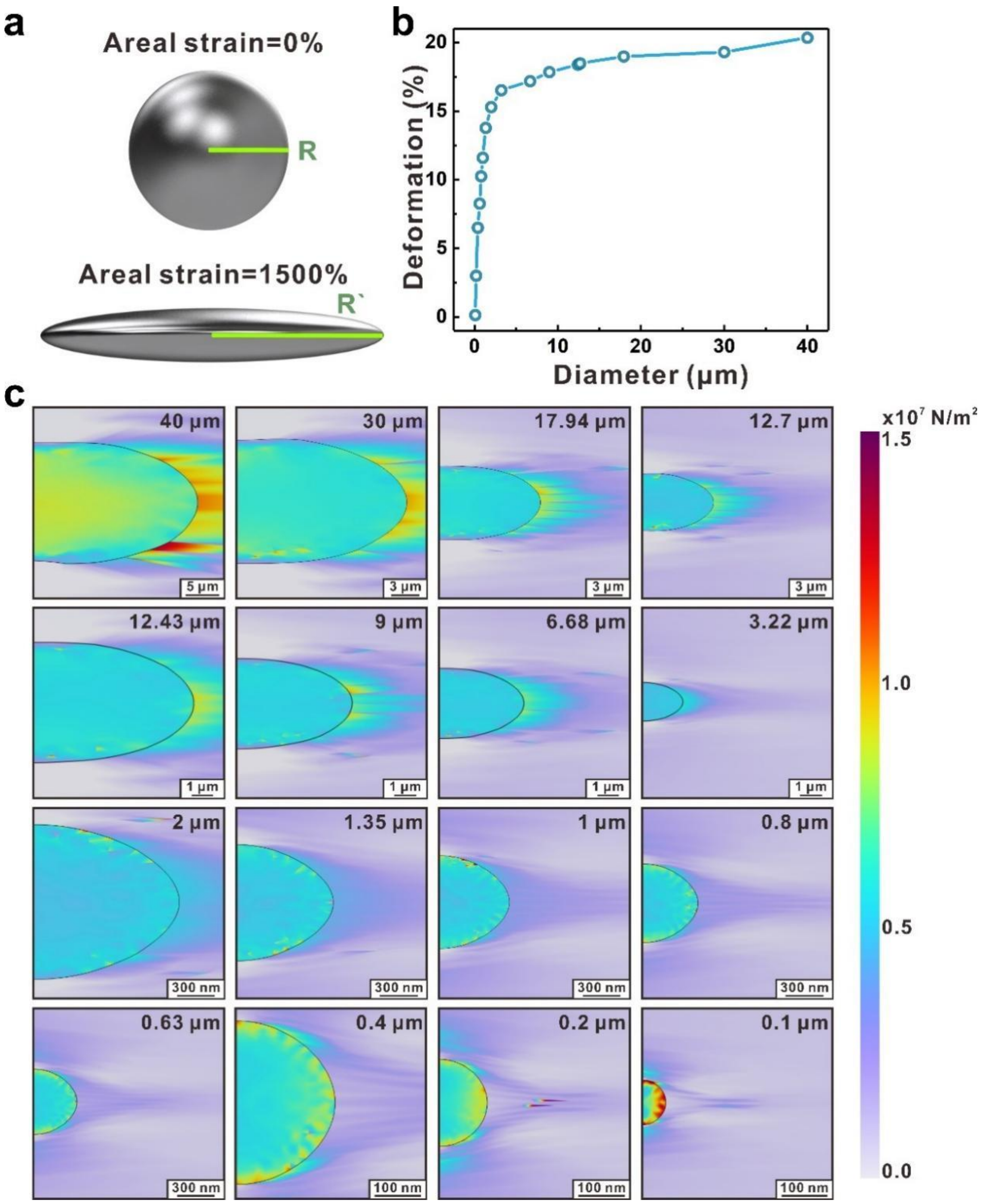

Supplementary Figure 11. Simulated deformation of liquid metal droplets. a Schematic illustration of liquid metal droplet with and without areal strain. **b** Calculated deformations of liquid metal droplets with different droplet sizes. **c** Simulation results of liquid metal droplets with sizes from 0.1 μm to 40 μm when under applied areal strain of 1500%.

Supplementary Note: From the calculation results, we find that the deformation of liquid metal droplets reduces with the decreasing size of liquid metal droplets. Especially, when the size of droplet is below 3.22 μm, the deformation reduces rapidly with the decreasing size. The liquid metal droplet with size of 0.1 μm hardly deform under areal strain. Such size-dependent deformation of liquid metal droplets has been also observed in liquid metal-based flexible circuits[8].

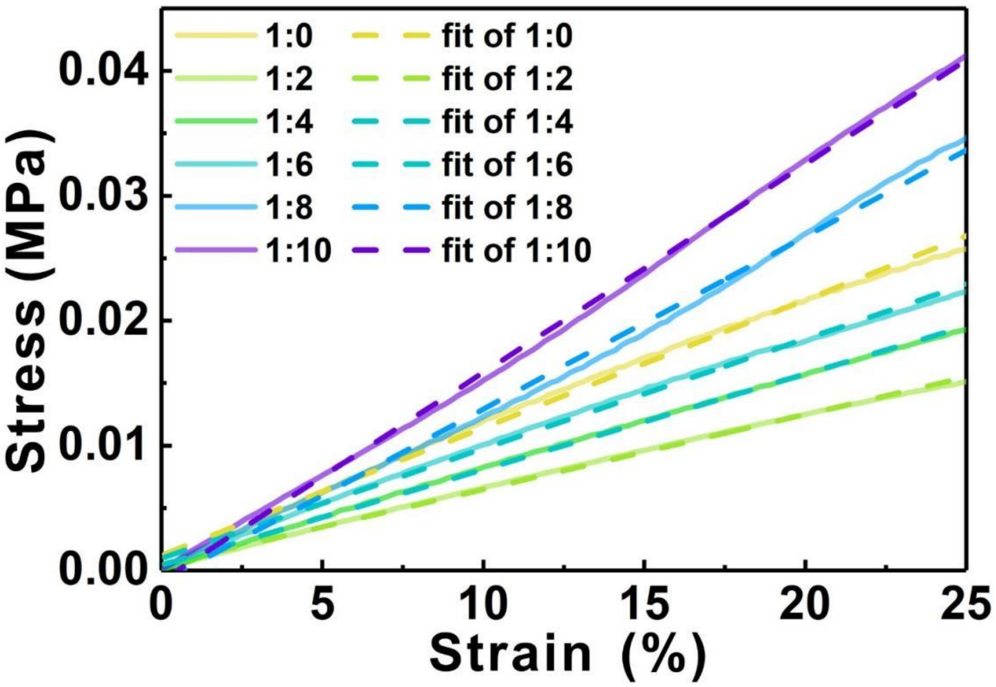

Supplementary Figure 12. Mechanical properties of BLEE films. The linear fitting stress-strain curves of BLEE films with different mass ratios of Ecoflex/EGaIn (1:0, 1:2, 1:4, 1:6, 1:8, and 1:10).

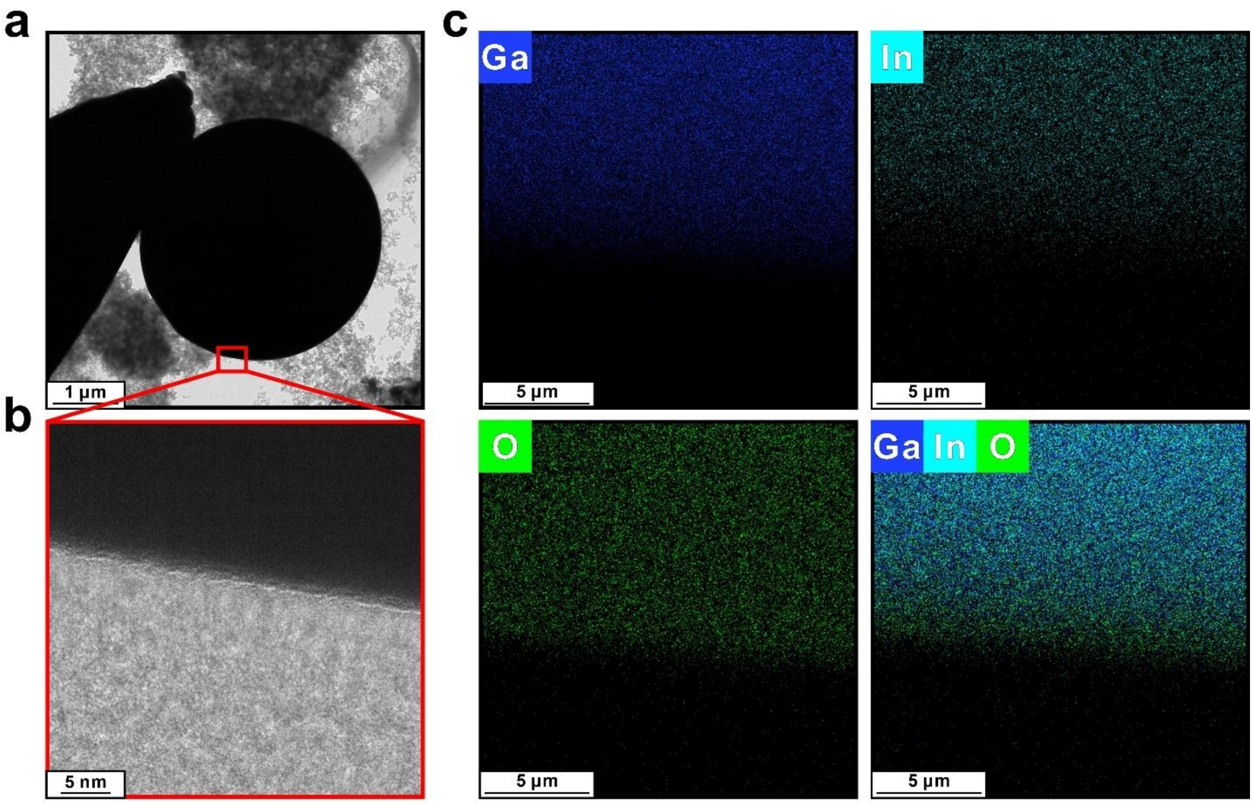

Supplementary Figure 13. TEM images of liquid metal droplets. a The TEM image (the scale bar is 1 μm), **b** high-resolution TEM image (the scale bar is 5 nm), and **c** energy dispersive spectrometer (EDS) mapping images of liquid metal droplets (the scale bar is 5 μm).

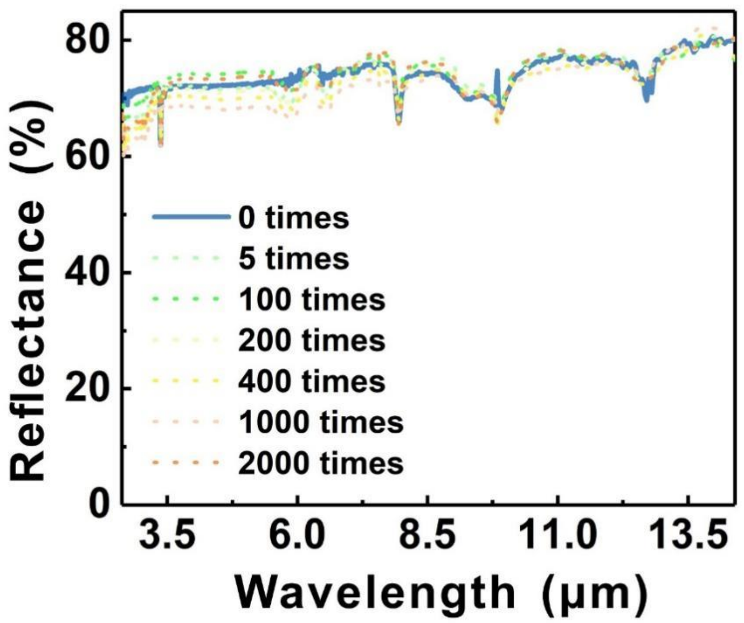

Supplementary Figure 14. Reliability of IR camouflage performance. The reflectance spectra of BLEE films with a mass ratio of Ecoflex/EGaIn (1:10) after applying an areal strain of 1500%. The data were taken after different cycles (0, 5, 100, 200, 40, 1000, and 2000).

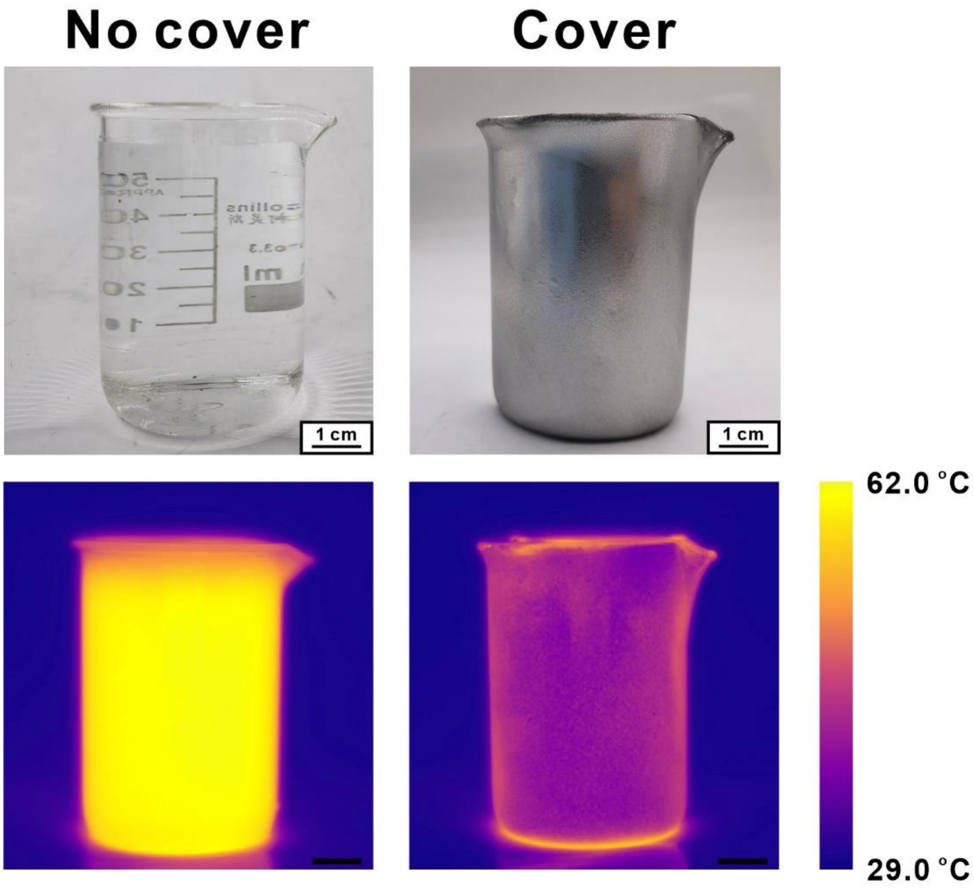

Supplementary Figure 15. IR camouflage of beaker. The optical and IR images of BLEE films with a mass ratio of Ecoflex/EGaIn (1:10) covering the beaker containing hot water under the areal strain of ~525%. The scale bar is 1 cm for both optical and IR images.

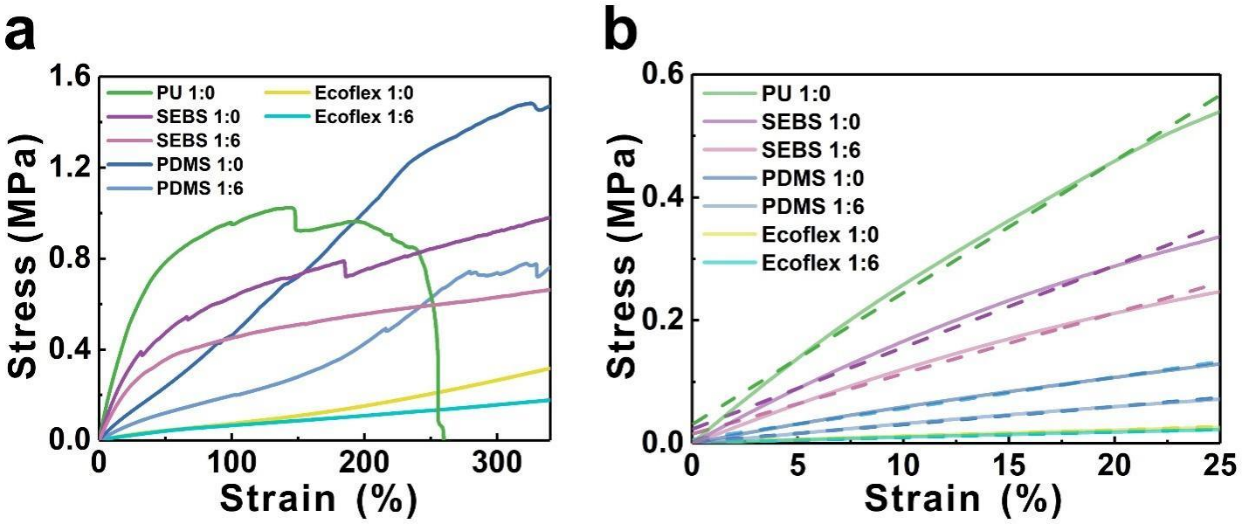

Supplementary Figure 16. Mechanical performance of different polymers. a The measured stress-strain curves, and **b** the linear fitting stress-strain curves (dashed lines) of BLEE films made from different polymers with mass ratios of 1:0 and 1:6.

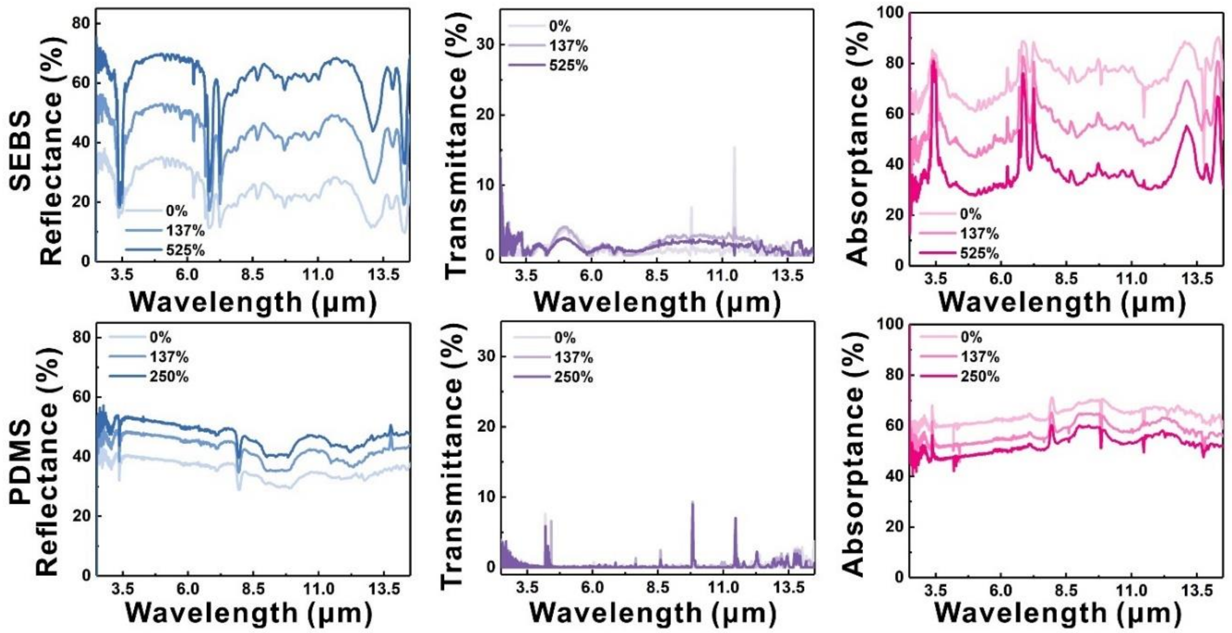

Supplementary Figure 17. The reflectance, transmittance, and absorptance spectra of BLEE films. The reflectance, transmittance, and absorptance spectra of BLEE films prepared by different polymer matrices after applying different areal strains (0%, 137%, 525% for SEBS, and 0%, 137%, 250% for PDMS).

Supplementary Note: To prove the generality of our method of fabricating liquid metal-based IR modulating materials, the other three kinds of elastomer materials (polyurethane (PU), styrene ethylene butylene styrene (SEBS), and polydimethylsiloxane (PDMS)) have been selected. The mechanical testing results (Supplementary Fig. 16 and Supplementary Table 4) show that the addition of liquid metal softened the composite, and pure PU could not be further mixed with liquid metal due to its poor mechanical performance. The IR specular reflectance results show that BLEE films prepared by either SEBS or PDMS shows mechanical stimulated IR-modulating reflectance (Supplementary Fig. 17).

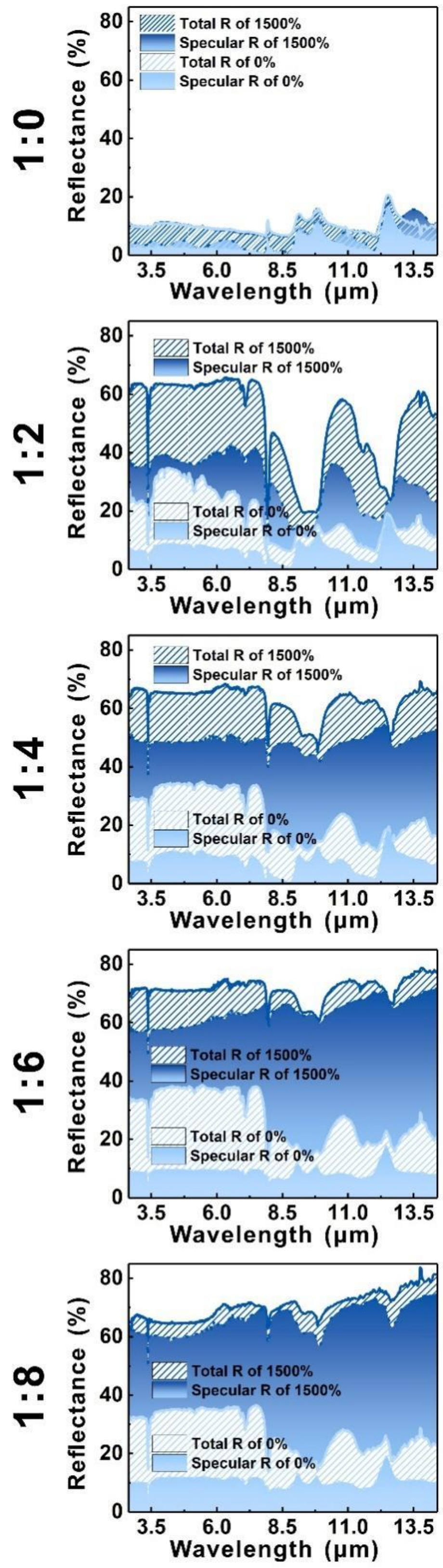

Supplementary Figure 18. The total and specular reflectance spectra of BLEE films. The total and specular reflectance spectra of BLEE films with different mass ratios of Ecoflex/EGaIn (1:0, 1:2, 1:4, 1:6, and 1:8) after applying areal strains of 0% and 1500%, respectively.

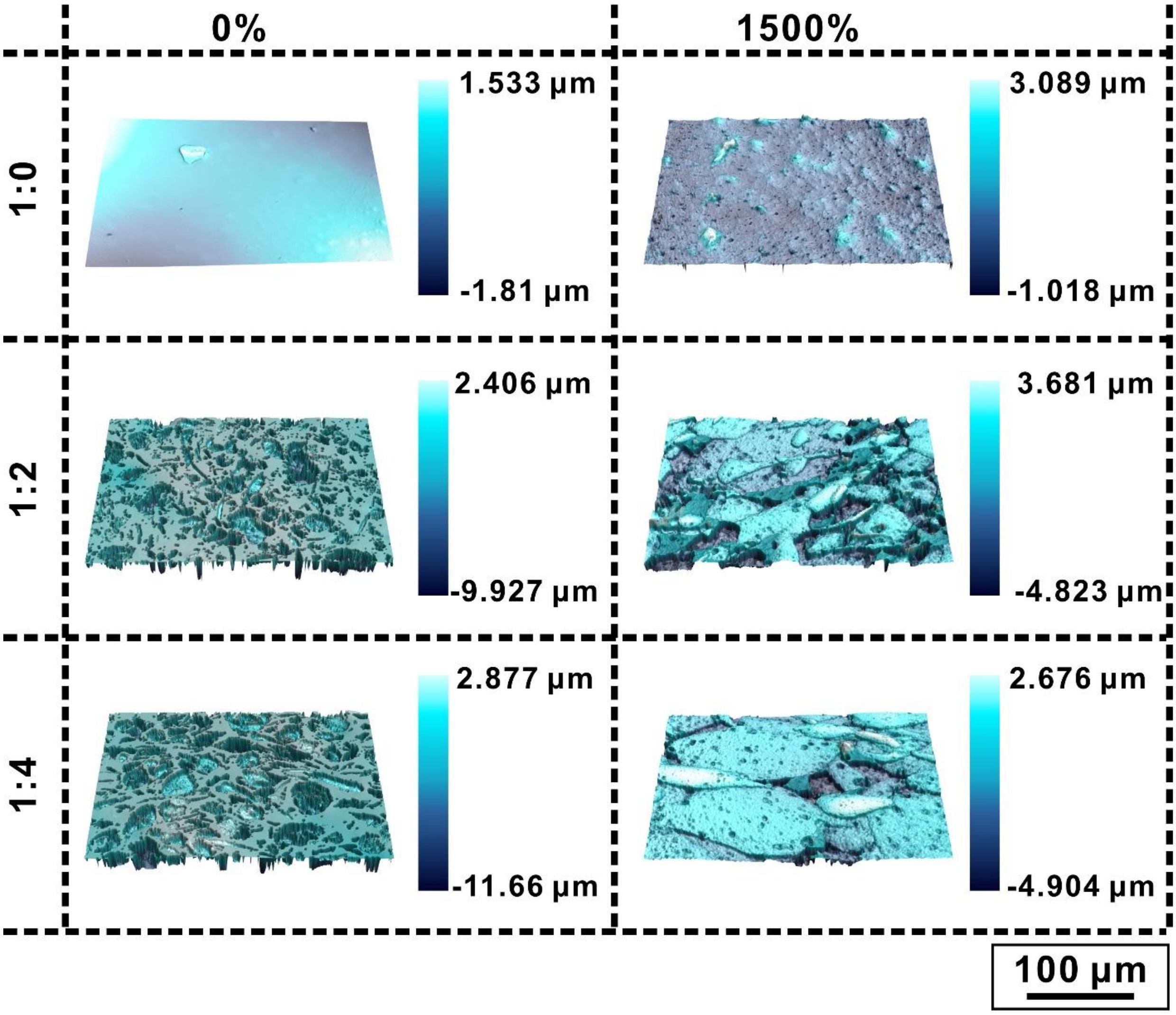

Supplementary Figure 19. The 3D surface topography images of BLEE films. The 3D surface topography images of BLEE films with mass ratios of 1:0, 1:2, and 1:4 after applying areal strains of 0% and 1500%, respectively. The scale bar reflects the intensity of surface fluctuation. The scale bar is 100 μm.

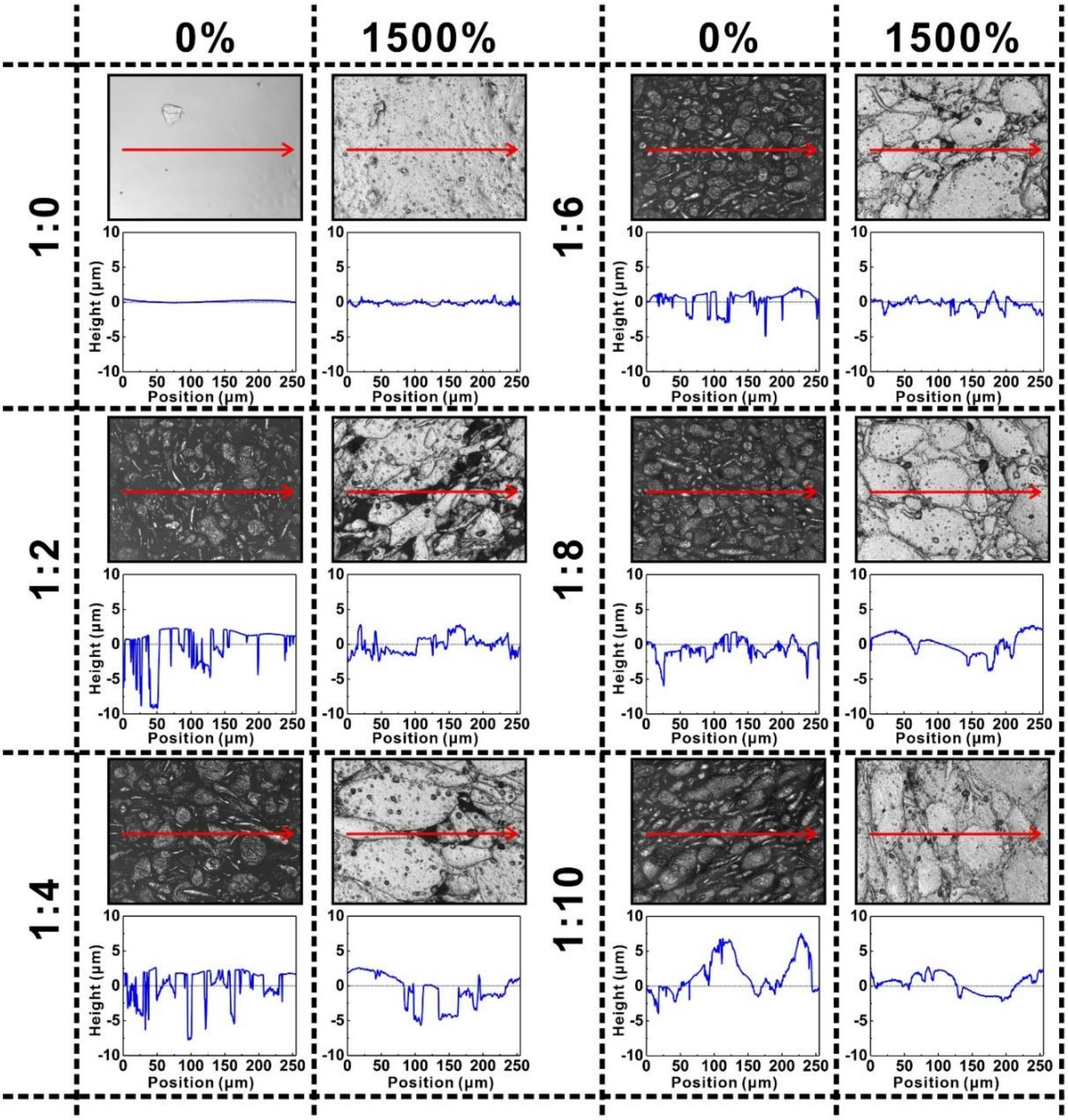

Supplementary Figure 20. The optical images and altitude variation of the surface of BLEE films. The optical images and altitude variation of the surface of BLEE films (mass ratios: 1:0, 1:2, 1:4, 1:6, 1:8, and 1:10) along the red line after applying areal strains of 0% and 1500%, respectively.

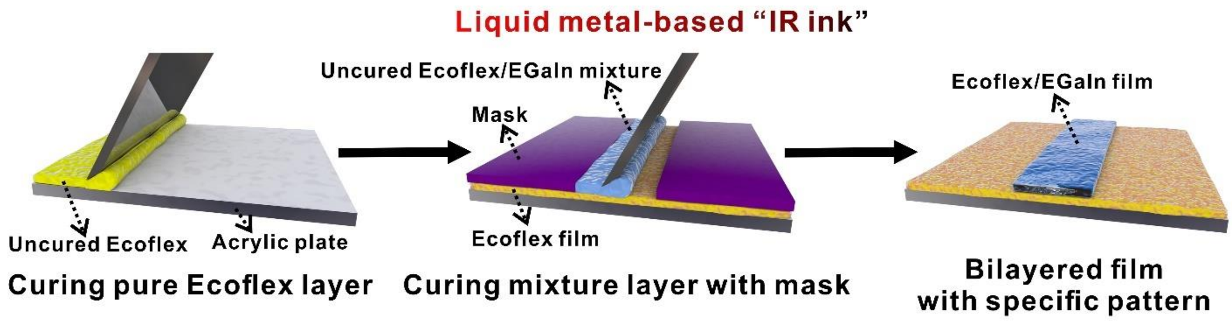

Supplementary Figure 21. Schematic illustration of the fabrication process of the "SJTU" pattern.

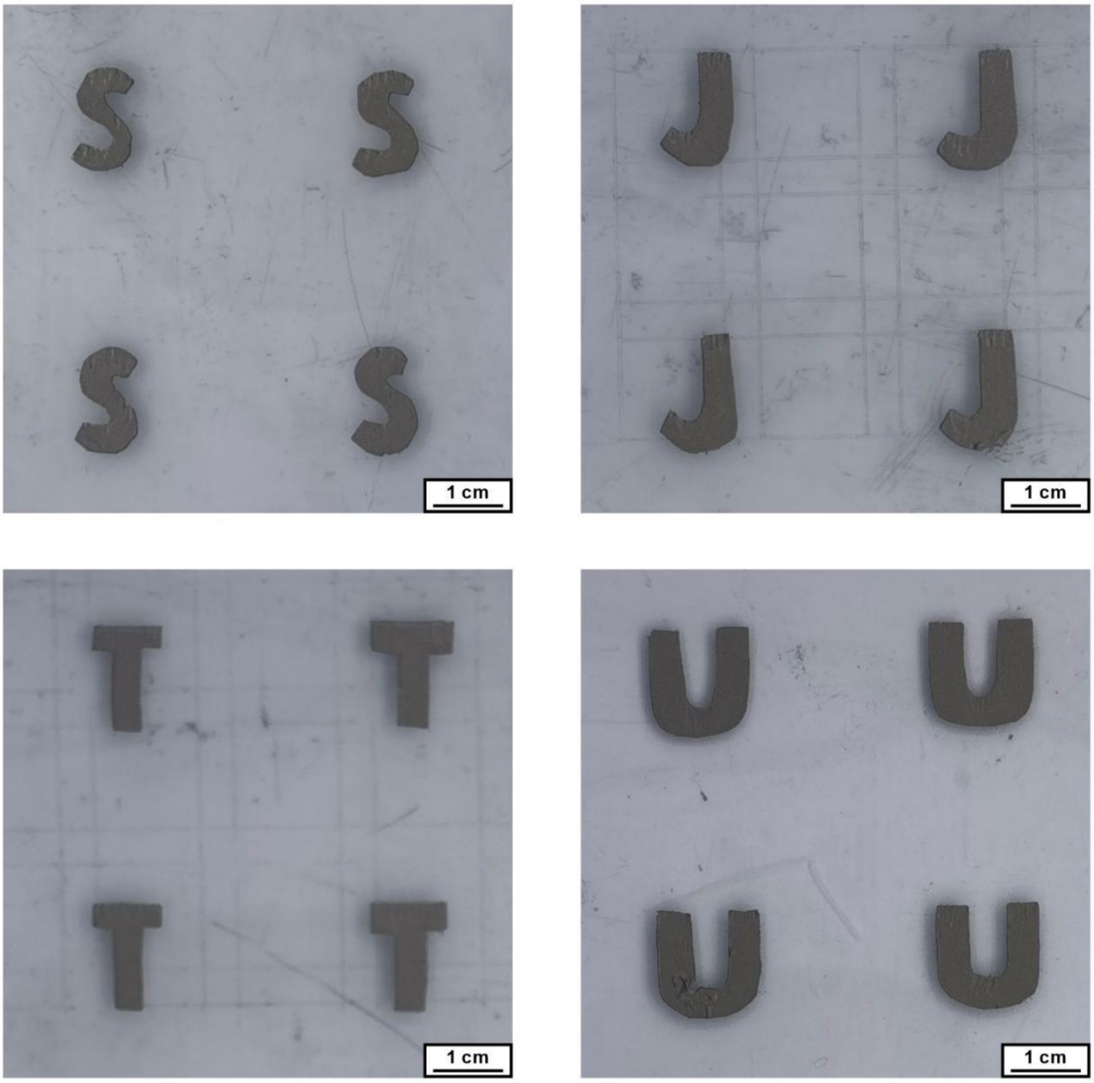

Supplementary Figure 22. The optical images of the as-prepared "SJTU" letter. The scale bar is 1 cm.

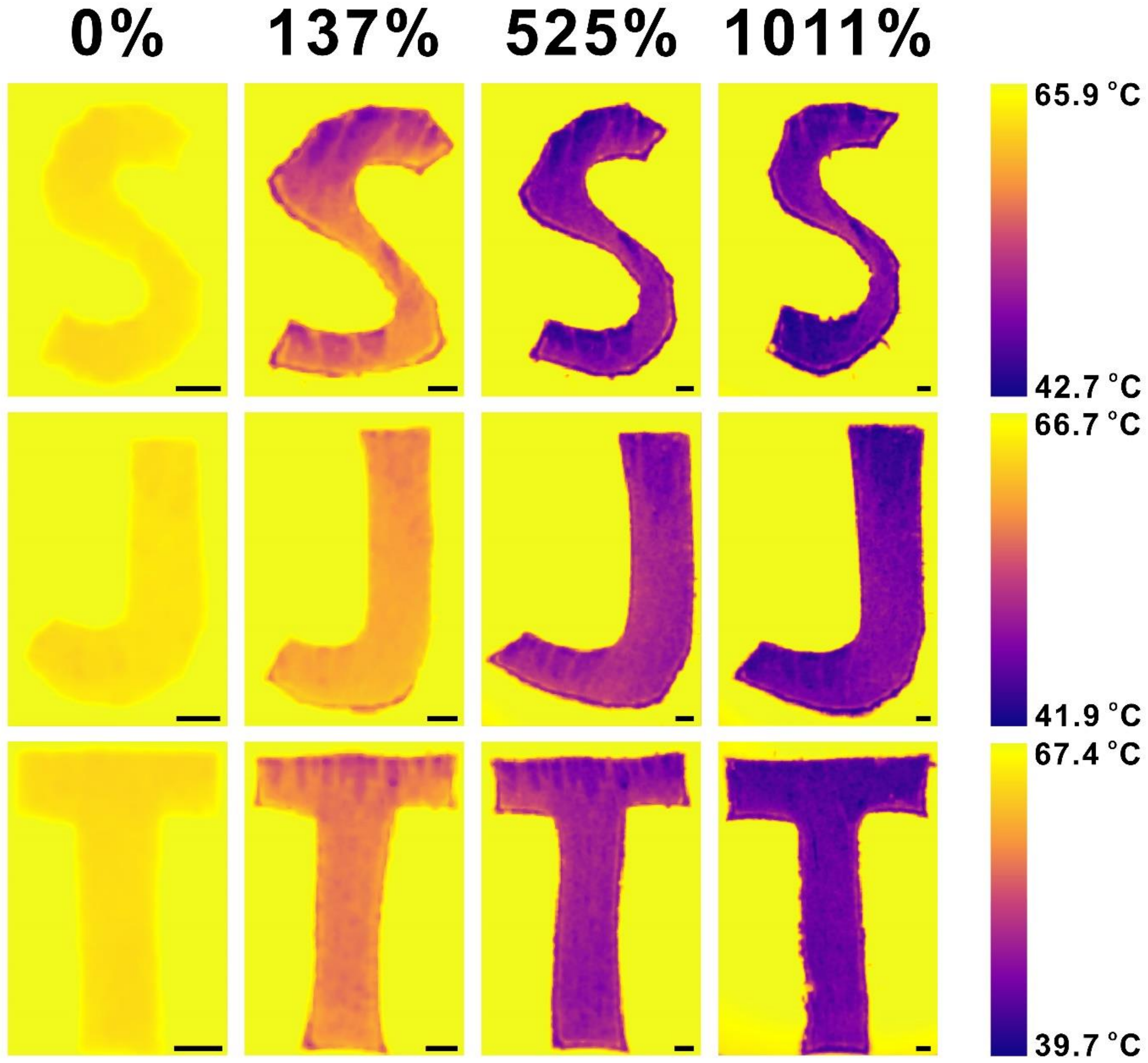

Supplementary Figure 23. IR images of the "S, J, T" pattern. IR images of the "S, J, T" pattern after applying different areal strains (0%, 137%, 525%, and 1011%). The scale bar is 0.25 cm.

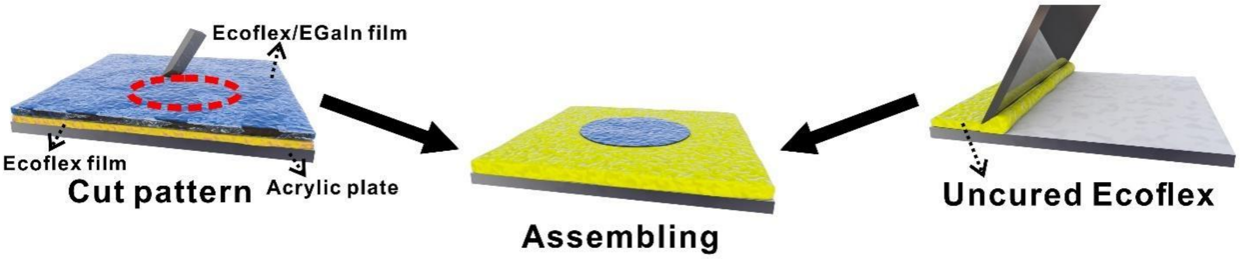

Supplementary Figure 24. Schematic illustration of the fabrication process of the "flower" pattern.

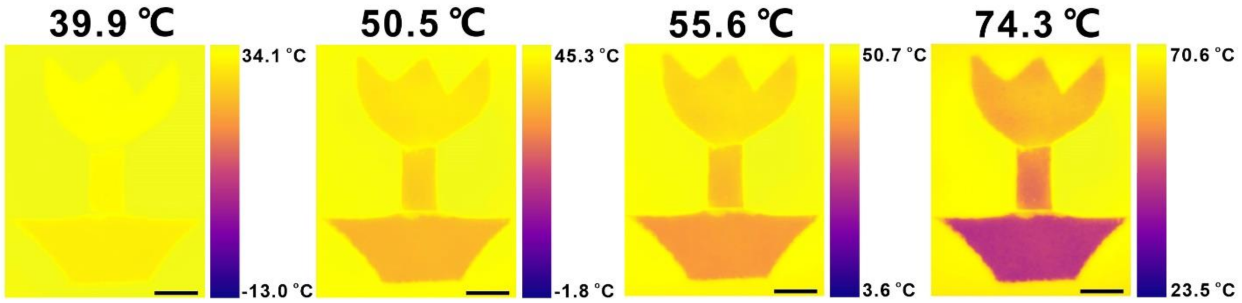

Supplementary Figure 25. The IR images of a "flower" pattern. The IR images of a "flower" pattern assembled from BLEE film parts with different mass ratios of Ecoflex/EGaIn. The samples were placed on the hot plate at different temperatures. The scale bar is 1 cm.

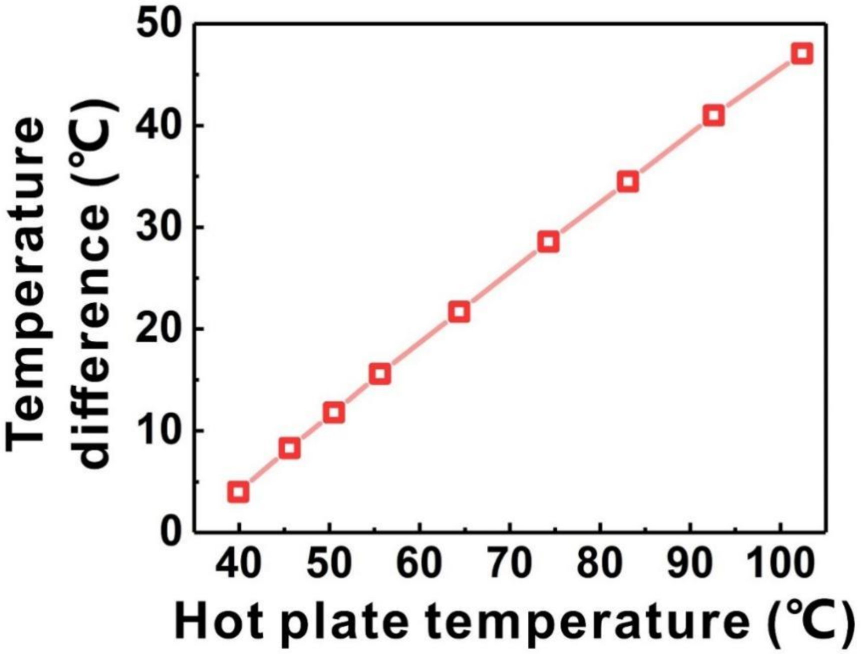

Supplementary Figure 26. The temperature difference of "flower" pattern. The difference between the highest temperature and the lowest temperature in the IR images of Fig. 4b and Supplementary Fig. 25 when the hot plate was set at different temperatures.

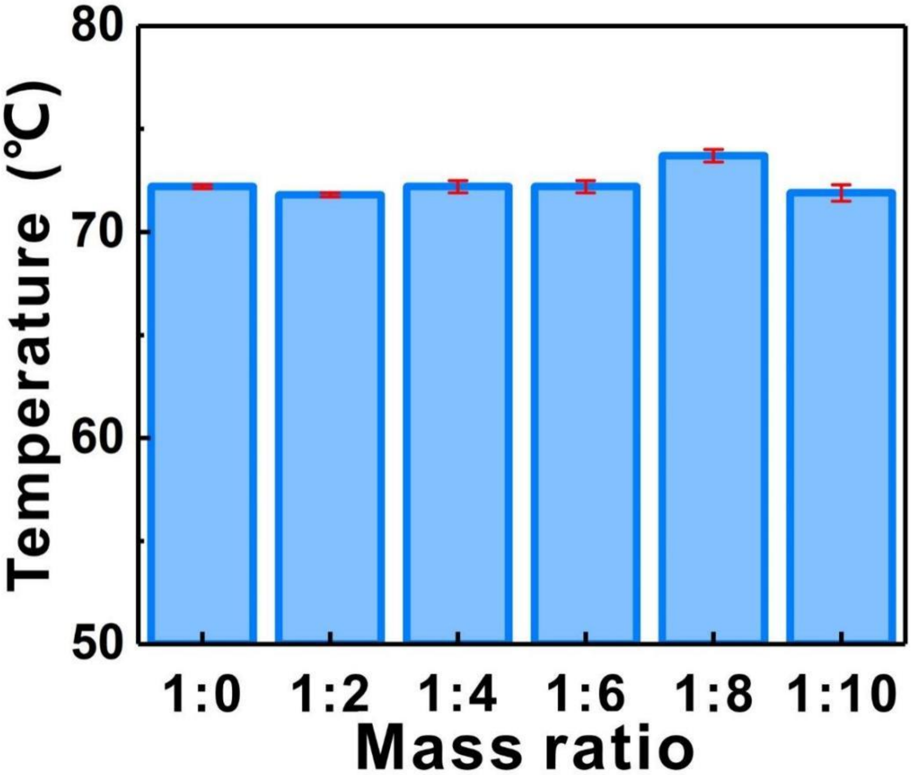

Supplementary Figure 27. The apparent temperature of bottom layer of BLEE films. The apparent temperature of bottom layer of BLEE films with different mass ratios of Ecoflex/EGaIn (1:0, 1:2, 1:4, 1:6, 1:8, and 1:10) when being placed on the hot plate without applying areal strain. All error bars represent the standard deviation.

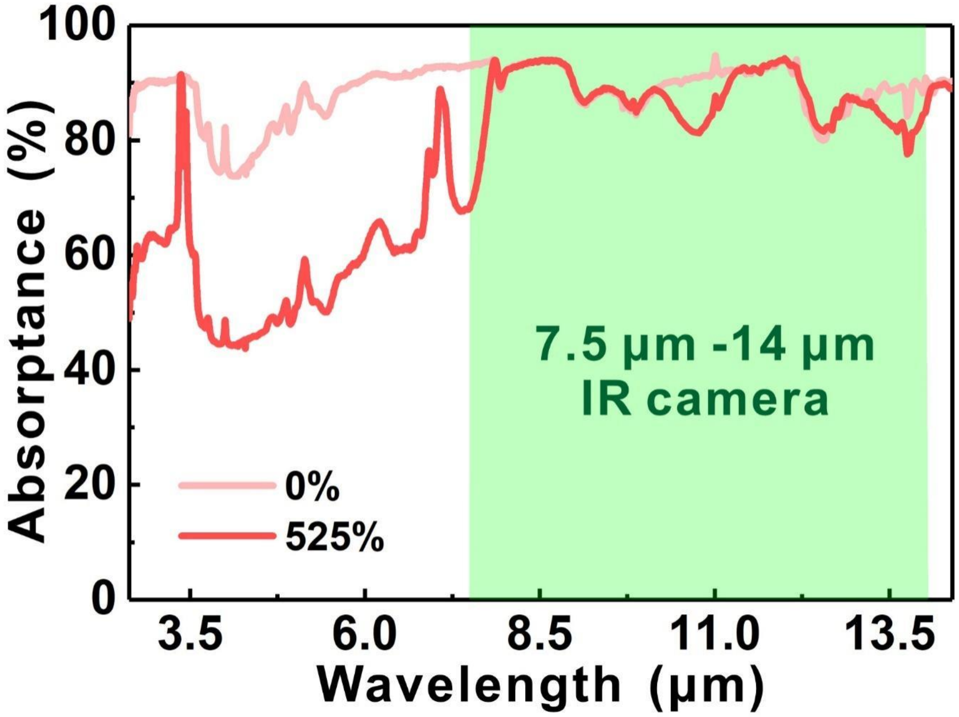

Supplementary Figure 28. The absorptance spectra of the bottom layer of BLEE film. The absorptance spectra of the bottom layer of BLEE film with a mass ratio of 1:10 when being applied areal strains of 0% and 525%, respectively. Green range represents the wavelength from 7.5 μm to 14 μm.

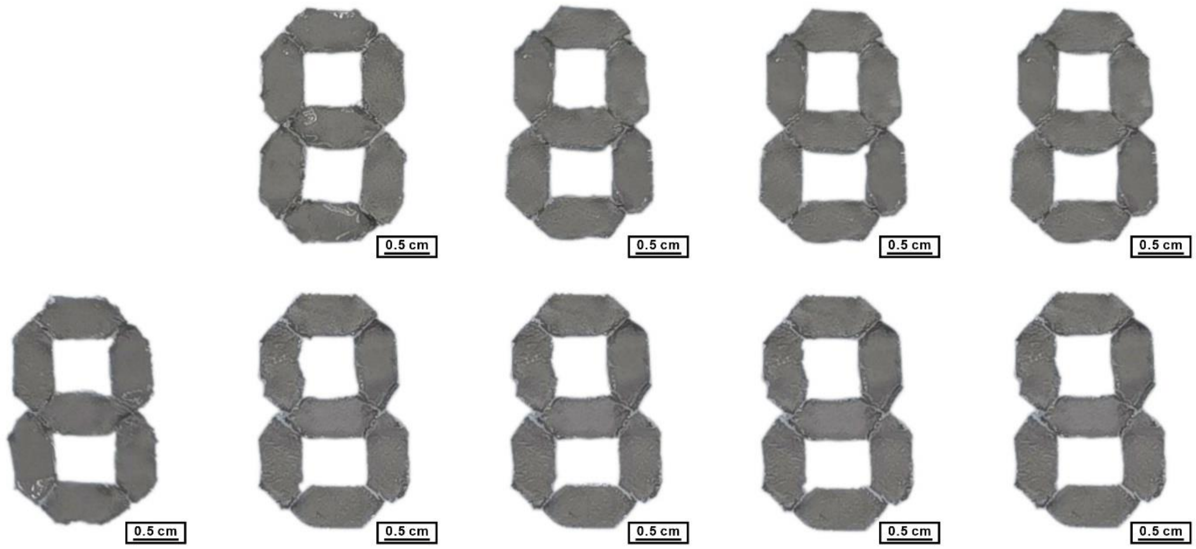

Supplementary Figure 29. The optical images of “numbers” assembled by BLEE films. The optical images of “numbers” assembled by BLEE films with a mass ratio of 1:10 for IR encoding/decoding before applying areal strain. (from top to bottom, from left to right: 1, 2, 3, 4, 5, 6, 7, 8, and 9, all the hidden numbers can be displayed by IR camera). The scale bar is 0.5 cm.

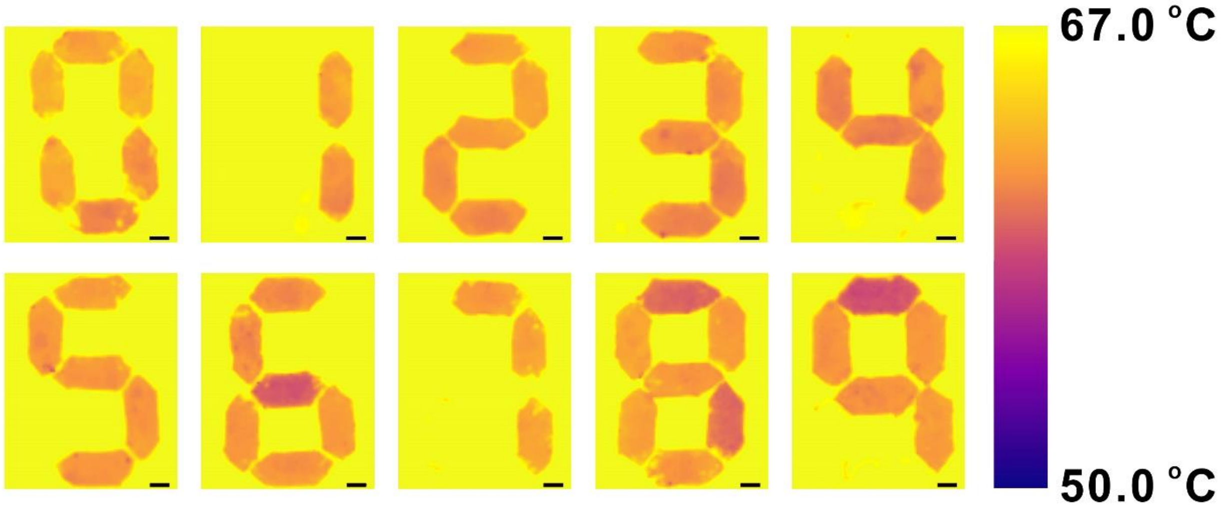

Supplementary Figure 30. The IR images of “numbers” assembled by BLEE films. The IR images of “numbers” assembled by BLEE films with mass ratio of 1:10 for IR encoding/decoding when being placed on the hot plate without applying areal strain. The scale bar is 0.25 cm.

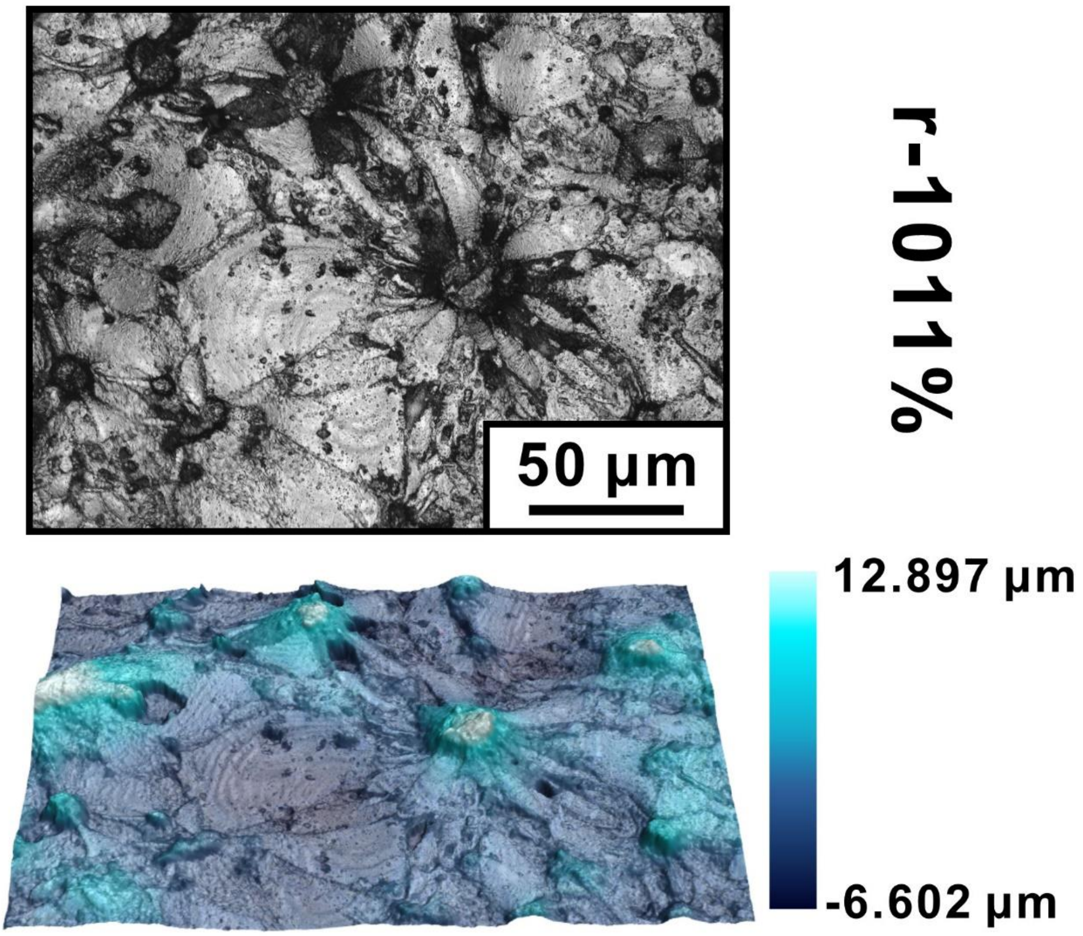

Supplementary Figure 31. The optical and 3D surface tomography images of the surface of the film (r-1011%). The scale bar reflects the intensity of surface fluctuation. And the scale bar is 50 μm.

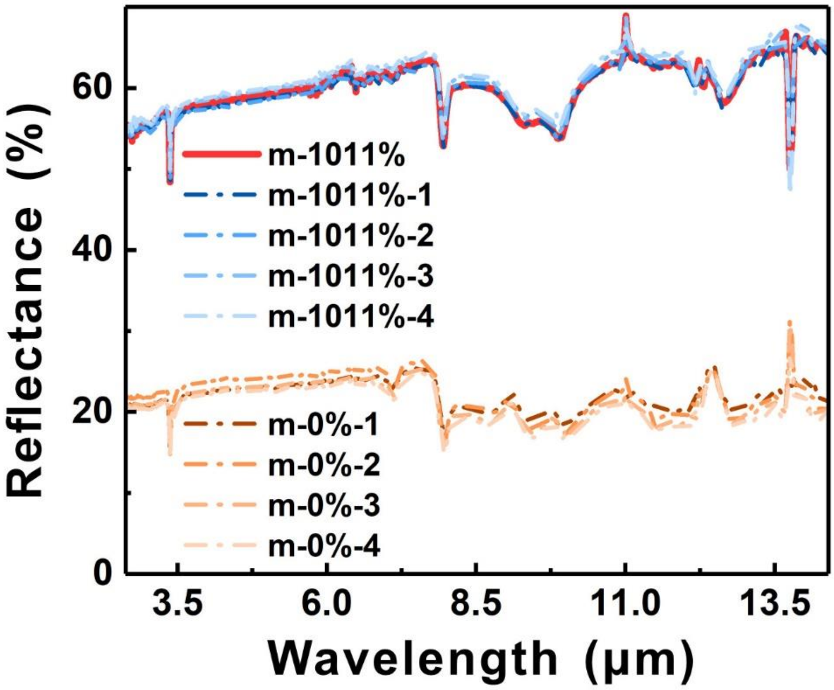

Supplementary Figure 32. The reflectance spectra of Ecoflex/low mp alloy (mp: 70 °C) films with and without applied areal strain after different cycles.

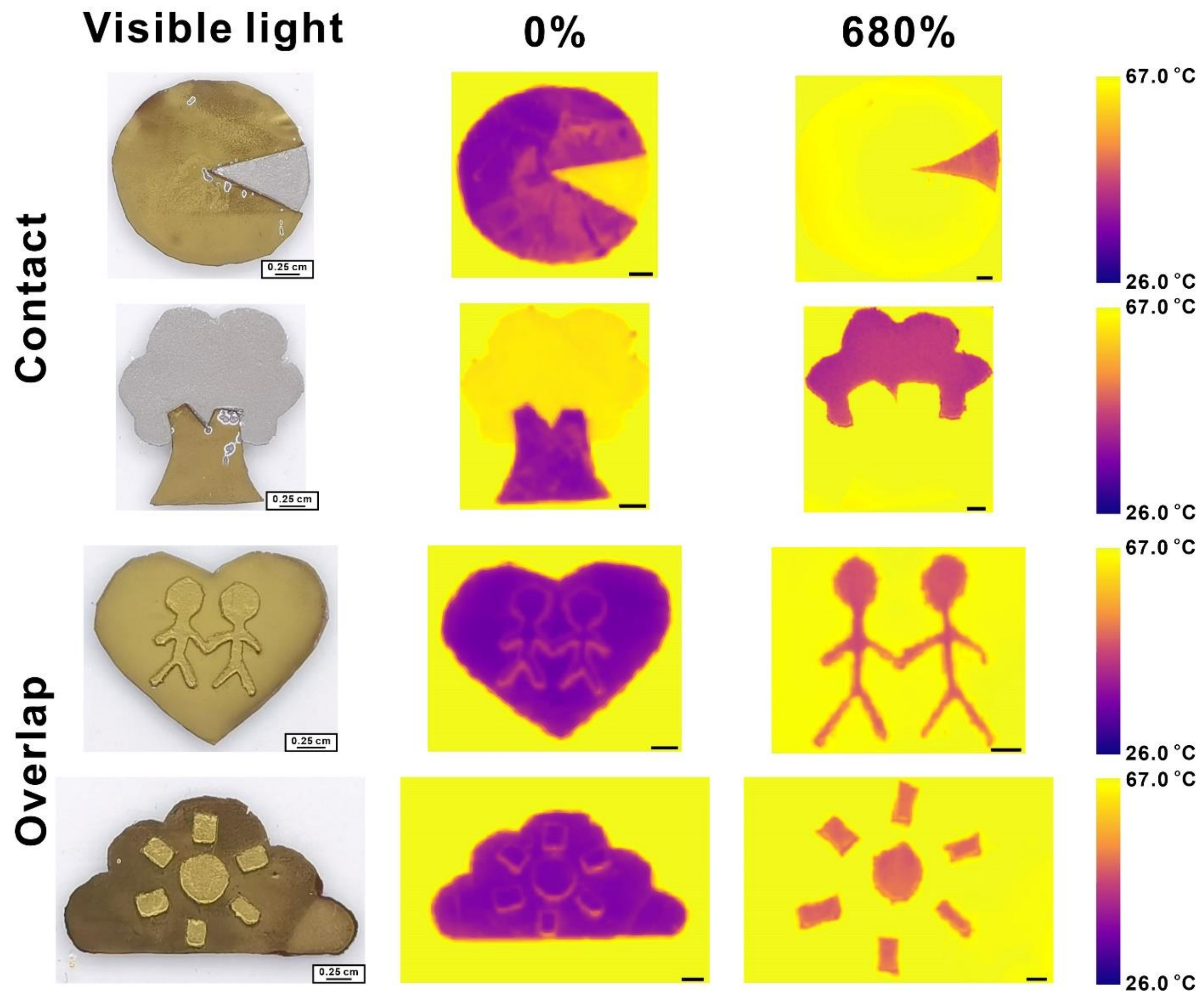

Supplementary Figure 33. The optical and IR images (before and after stretching) of the samples for multi-layered programmable IR encryption. The scale bar is 0.25 cm for optical images and IR images (before stretching). And the scale bar is 0.5 cm for IR images (after stretching).

Supplementary Note 1: As expected in contact mode, the IR pattern made of gold (Au) film was visible without stretching because of high reflectance. After applying areal strain, the pattern made from BLEE film showed high IR reflectance.

Supplementary Note 2: As for overlap mode, the BLEE layer was fully covered by evaporated Au, and the pattern made from BLEE film was hiding in the Au film. After the sample was mechanically stretched, the pattern made from Au film disappeared and the pattern made from BLEE film appeared in the IR image.

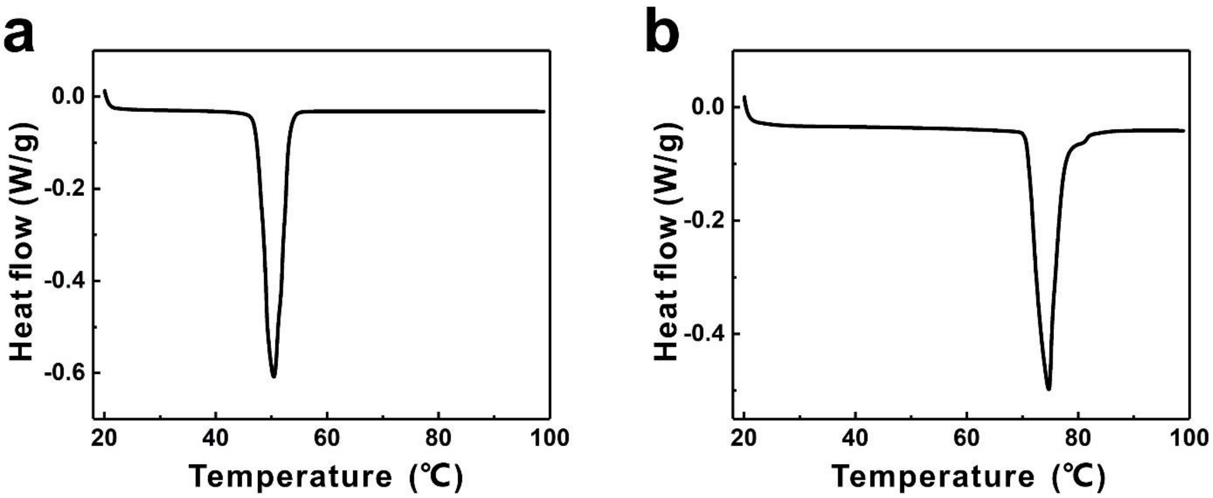

Supplementary Figure 34. The differential scanning calorimeter (DSC) curves of Ecoflex/low mp alloy. The differential scanning calorimeter (DSC) curves show the melting process of Ecoflex/low mp alloy (mp at **a** 47 ℃. and **b** 70 ℃) films. From the DSC results, the mp of films is 48.0 ℃ and 70.9 ℃, respectively.

Supplementary Table 1. The temperature of BLEE films with different mass ratios of Ecoflex/EGaIn (1:0, 1:2, 1:4, 1:6, 1:8, and 1:10) on the hot plate taken from IR images, when applying different areal strains (0%, 137%, 525%, 1011%, and 1500%). All error bars represent the standard deviation.

Samples	1:0	1:2	1:4	1:6	1:8	1:10
0% (℃)	72.2±0.1	70.1±0.2	70.1±0.1	68.6±0.2	65.9±0.1	63.3±0.1
137% (℃)	72.6±0.1	67.4±0.6	64.2±0.5	62.1±0.4	60.3±0.4	57.3±0.5
525% (℃)	72.6±0.1	63.9±1.0	56.8±0.9	50.9±0.9	49.0±0.4	45.9±0.5
1011% (℃)	72.3±0.1	61.2±1.7	52.9±0.9	45.1±0.7	42.6±0.6	41.9±0.7
1500% (℃)	72.5±0.1	60.2±2.0	52.1±1.1	44.3±0.5	41.9±0.2	41.5±0.5

Supplementary Table 2. The temperature calculated from the reflectance spectra of BLEE films with different mass ratios of Ecoflex/EGaIn (1:0, 1:2, 1:4, 1:6, 1:8, and 1:10) on the hot plate, when applying different areal strains (0%, 137%, 525%, 1011%, and 1500%).

Samples	1:0	1:2	1:4	1:6	1:8	1:10
0% (℃)	72.46	71.76	69.51	67.84	67.15	64.02
137% (℃)	72.90	68.27	64.17	63.28	61.69	55.50
525% (℃)	72.22	64.19	56.18	49.48	48.79	46.47
1011% (℃)	72.52	61.60	52.75	47.36	44.98	43.55
1500% (℃)	72.34	58.97	49.93	44.40	43.74	42.33

Supplementary Table 3. Young's moduli of BLEE films with different mass ratios of Ecoflex/EGaIn (1:0, 1:2, 1:4, 1:6, 1:8, and 1:10) are calculated from Supplementary Fig. 12.

Samples	1:0	1:2	1:4	1:6	1:8	1:10
Young's Modulus (MPa)	0.102	0.060	0.077	0.088	0.138	0.167

Supplementary Table 4. Young's moduli of different BLEE films prepared with different mass ratios of 1:0 and 1:6, which is calculated from Supplementary Fig. 16b. (Young's moduli of Ecoflex were taken from Table 3)

Samples	PU 1:0	SEBS 1:0	SEBS 1:6	PDMS 1:0	PDMS 1:6	Ecofelx 1:0	Ecofelx 1:6
Young's modulus (MPa)	2.142	1.330	0.985	0.290	0.508	0.102	0.088

Supplementary Table 5. The surface roughness of BLEE films with different mass ratios of Ecoflex/EGaIn (1:0, 1:2, 1:4, 1:6, 1:8, and 1:10), when applying different areal strains (0% and 1500%).

Samples	1:0	1:2	1:4	1:6	1:8	1:10
0% (μm)	0.518	2.072	2.212	1.175	1.311	2.506
1500% (μm)	0.455	1.436	0.916	0.920	1.022	1.030

Supplementary Note 1: Without applying areal strain, small amounts of EGaIn droplets dispersed in the polymer cause surface roughness. The roughness increases with the portion of EGaIn when the mass ratio is 1:2 or 1:4. When the mass ratio is 1:6, the amount of EGaIn droplets is enough to fill up the surface and the size of the droplets decreases (Supplementary Figs. 1 and 6)[9,10]. Therefore, the roughness drops sharply. When the mass ratio is 1:8 or 1:10, EGaIn droplets stack on each other, leading to a dramatic increase in roughness.

Supplementary Note 2: After applying areal strain, the scattered EGaIn droplets in the polymer (mass ratio of 1:2) are similar to the bumps of surface, resulting in large roughness. Under stretch, the EGaIn droplets (mass ratio of 1:4) fill in the gaps between each other in the polymer and reduce the surface roughness. When the mass ratio is 1:6, 1:8, or 1:10, the EGaIn droplets begin to overlap with each other in polymer, which slightly increases the surface roughness.

Supplementary Table 6. The measured apparent temperature of Ecoflex/low mp alloy (mp: 70 ℃ (Sn: 25-30 wt.%; Bi: 50-60 wt.%; and Pb: 20-25 wt.%)) films. All error bars represent the standard deviation.

Samples	r-0%	r-1011%	m-1011%
64 ℃	56.2±0.3	42.2±0.5	39.5±0.4
104 ℃	90.4±0.5	66.0±0.7	60.6±1.1

Supplementary Table 7. The calculated apparent temperature from the reflectance spectra of Ecoflex/low mp alloy (mp: 70 °C) films.

Samples	r-0%	r-1011%	m-1011%
64 °C	56.20	43.7	40.76
104 °C	89.30	65.66	60.91

Supplementary Table 8. The surface roughness of different Ecoflex/low mp alloy (mp: 70 °C) films.

Samples	r-0%	r-1011%	m-1011%
Surface roughness (μm)	1.774	2.874	1.294

Supplementary Table 9. The thickness of single-layered films and BLEE films with different mass ratios of Ecoflex/EGaIn (1:0, 1:2, 1:4, 1:6, 1:8, and 1:10). All error bars represent the standard deviation.

Samples	1:0	1:2	1:4	1:6	1:8	1:10
Single-layered (μm)	100.0	114.0±1.0	113.7±1.2	120.3±3.8	126.0±4.4	122.0±4.4
Bilayered (μm)	/	127.3±2.5	133.0±1.0	121.7±1.5	132.0±1.0	136.3±1.5

Supplementary Table 10. The maximum and minimum temperature values of the scale bar for the "SJTU" pattern samples.

Sample	S	J	T	U
Maximum (℃)	65.9	66.7	67.4	65.6
Minimum (℃)	42.7	41.9	39.7	42.6

Supplementary Table 11. The maximum and minimum temperature values of the scale bar for the "flower" pattern samples placed on the hot plate of different temperatures.

Maximum (℃)	100.4	90.0	79.9	70.6	60.0	50.7	45.3	40.1	34.1
Minimum (℃)	53.3	42.9	32.8	23.5	12.9	3.6	-1.8	-7.0	-13.0

Supplementary References

1. Liu, Y., Feng, Z., Xu, C., Chatterjee, A. & Gorodetsky, A. A. Reconfigurable Micro- And Nano-Structured Camouflage Surfaces Inspired by Cephalopods. *ACS Nano* **15**, 17299–17309 (2021).
2. Holman, J. P., *Heat Transfer* (McGraw-hill, 1986).
3. Boonvisut, P., Jackson, R. & Çavuşoğlu, M. C. Estimation of soft tissue mechanical parameters from robotic manipulation data. *Proc. - IEEE Int. Conf. Robot. Autom.* 4667–4674 (2012) doi:10.1109/ICRA.2012.6225071.
4. Style, R. W., Tutika, R., Kim, J. Y. & Bartlett, M. D. Solid–Liquid Composites for Soft Multifunctional Materials. *Adv. Funct. Mater.* **31**, (2021).
5. Catalán-Gómez, S. *et al.* Modification of the Mechanical Properties of Core-Shell Liquid Gallium Nanoparticles by Thermal Oxidation at Low Temperature. *Part. Part. Syst. Charact.* **38**, 1–8 (2021).
6. Farrell, Z. J. & Tabor, C. Control of Gallium Oxide Growth on Liquid Metal Eutectic Gallium/Indium Nanoparticles via Thiolation. *Langmuir* **34**, 234–240 (2018).
7. Fan, P. *et al.* Nano liquid metal for the preparation of a thermally conductive and electrically insulating material with high stability. *RSC Adv.* **8**, 16232–16242 (2018).
8. Lee, W. *et al.* Universal assembly of liquid metal particles in polymers enables elastic printed circuit board. *Science (80-.).* **378**, 637–641 (2022).
9. Shah, N. U. H. *et al.* Gallium oxide-stabilized oil in liquid metal emulsions. *Soft Matter* **17**, 8269–8275 (2021).

10. Neumann, T. V., Facchine, E. G., Leonardo, B., Khan, S. & Dickey, M. D. Direct write printing of a self-encapsulating liquid metal-silicone composite. *Soft Matter* **16**, 6608–6618 (2020).
