# Supplementary material for: Chameleon-inspired tunable multi-layered infrared-modulating system via stretchable liquid metal microdroplets in elastomer film
Source: Nat Commun. 2024 Jun 26;15:5395. doi: 10.1038/s41467-024-49849-y (PMC11208509; doi:10.1038/s41467-024-49849-y)
Supplement: Supplementary file 4 — Description of Additional Supplementary Files [file 41467_2024_49849_MOESM4_ESM.pdf]

Description of Additional Supplementary Files

File Name: Supplementary Movie 1

Description: 3D reconstructor analysis of BLEE (1:2- 0%).

File Name: Supplementary Movie 2

Description: 3D reconstructor analysis of BLEE (1:2- 1500%).

File Name: Supplementary Movie 3

Description: 3D reconstructor analysis of BLEE (1:10- 0%).

File Name: Supplementary Movie 4

Description: 3D reconstructor analysis of BLEE (1:10- 1500%).

File Name: Supplementary Movie 5

Description: Decoding hidden numbers using IR camera.
